# Light‐Based Juxtacrine Signaling Between Synthetic Cells

**DOI:** 10.1002/smsc.202400401

**Published:** 2024-10-30

**Authors:** Hossein Moghimianavval, Kyle J. Loi, Sung‐Won Hwang, Yashar Bashirzadeh, Allen P. Liu

**Affiliations:** ^1^ Department of Mechanical Engineering University of Michigan Ann Arbor MI 48109 USA; ^2^ Neuroscience Program University of Michigan Ann Arbor MI 48109 USA; ^3^ Cellular and Molecular Biology Program University of Michigan Ann Arbor MI 48109 USA; ^4^ Department of Chemical Engineering University of Michigan Ann Arbor MI 48109 USA; ^5^ Department of Biomedical Engineering University of Michigan Ann Arbor MI 48109 USA; ^6^ Department of Biophysics University of Michigan Ann Arbor MI 48109 USA

**Keywords:** juxtacrine signalings, light‐induced dimerizing proteins (iLIDs)–SspB, Nanoluc Binary Technology (NanoBiT), SpyTag–SpyCatcher, synthetic cell communications, synthetic cells

## Abstract

Cell signaling through direct physical cell–cell contacts plays vital roles in biology during development, angiogenesis, and immune response. Intercellular communication mechanisms between synthetic cells constructed from the bottom up are majorly reliant on diffusible chemical signals, thus limiting the range of responses in receiver cells. Engineering contact‐dependent signaling between synthetic cells promises to unlock more complicated signaling schemes with spatial responses. Herein, a light‐activated contact‐dependent communication scheme for synthetic cells is designed and demonstrated. A split luminescent protein is utilized to limit signal generation exclusively to contact interfaces of synthetic cells, driving the recruitment of a photoswitchable protein in receiver cells, akin to juxtacrine signaling in living cells. The modular design not only demonstrates contact‐dependent communication between synthetic cells but also provides a platform for engineering orthogonal contact‐dependent signaling mechanisms.

## Graphical abstract

A juxtacrine signaling mechanism between synthetic cells that are in direct physical contact expands the potential capabilities of synthetic cells as biomaterials and synthetic immune cells. Here, light‐based juxtacrine signaling based on SpyTag–SpyCatcher‐mediated reconstitution of a split luciferase, Nanoluc Binary Technology, in the interface of two synthetic cells is demonstrated to drive light‐activated dimerization of light‐induced dimerizing protein (iLID) and stringent starvation protein B.

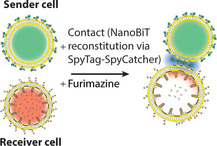

## 1 Introduction

Intercellular communication is a key characteristic of multicellular life. Cells utilize various intercellular communication mechanisms for exchanging information regarding their state, the density of their cellular community, and the presence of pathogens or competing cells.^[^ ^1^, ^2^ ^]^ While communication through diffusible chemicals is the primary means of signaling in prokaryotes,^[^ ^3^ ^]^ higher‐level organisms like metazoans have amassed a wide range of sophisticated communication mechanisms for short‐ and long‐distance signaling.^[^ ^4^ ^]^ Specifically, contact‐dependent or juxtacrine communication has emerged during evolution as a mechanism for highly specific and targeted communication, especially when signaling results in dramatic responses.^[^ ^5^, ^6^ ^]^ Notch–Delta signaling is an example of contact‐dependent communication with essential roles in gene regulation during development.^[^ ^7^, ^8^ ^]^ In Notch signaling, direct cell–cell contact enables protein–protein interaction at the contact interface of two cells. Upon interaction between the extracellular domains of Notch and Delta, the intracellular domain of Notch in the receiving cell is proteolytically released which functions to regulate transcription. Similar to Notch signaling, other processes such as T‐cell activation^[^ ^9^, ^10^ ^]^ and signaling through tight junctions^[^ ^11^ ^]^ and gap junctions^[^ ^12^ ^]^ rely on cell–cell interface formation and juxtaposed protein–protein interactions for signaling.

Reconstitution of intercellular communication in synthetic cells has recently gained increasing interest.^[^ ^13^, ^14^, ^15^ ^]^ The development of synthetic cells with functionalities ranging from light‐driven energy generation^[^ ^16^ ^]^ to biocomputing^[^ ^17^, ^18^ ^]^ and biosensing^[^ ^19^ ^]^ has propelled the capabilities of synthetic cells in various applications such as drug delivery and energy regeneration. Therefore, many recent endeavors have focused on implementing communication mechanisms in synthetic cells to enable engineering smart synthetic multicellular systems and synthetic cell–natural cell communication for therapeutic purposes.^[^ ^20^ ^]^ While initial development in synthetic cell communication utilized the release of membrane‐permeable chemical inducers from sender cells to regulate gene expression in receiver cells,^[^ ^21^, ^22^, ^23^, ^24^, ^25^ ^]^ more sophisticated mechanisms relying on membrane pores,^[^ ^26^, ^27^, ^28^ ^]^ clustered regularly interspaced short palindromic repeats‐Cas systems,^[^ ^29^ ^]^ or signal amplification^[^ ^30^ ^]^ were later developed. Further, the inclusion of light‐sensitive elements in the signaling cascade has enabled light‐assisted communication among both communities of synthetic cells as well as synthetic and natural cells.^[^ ^31^, ^32^, ^33^, ^34^, ^35^, ^36^ ^]^ However, previous studies such as Chakraborty et al.^[^ ^32^ ^]^ and Smith et al.^[^ ^31^ ^]^ use light to initialize signaling by creating adhesion between synthetic cells or driving cell‐free expression of an enzyme, respectively. Therefore, the signaling in these studies is controlled by light but occurs through the transport of chemical molecules, Ca^2+^ and N‐isovaleryl‐L‐homoserine lactone, respectively. To our knowledge, synthetic cell signaling with light as the *primary* signal where light is generated exclusively at the cell–cell contact interface has not been demonstrated to date.

A few synthetic cell contact‐dependent communication systems have been engineered previously,^[^ ^26^, ^37^, ^38^, ^39^ ^]^ although mostly in a network of connected aqueous droplets in oil. Such studies have relied on membrane pores such as α‐hemolysin for chemical communication between adjacent synthetic cells. This has effectively limited the communication between synthetic cells to chemical signaling. The development of SynNotch in natural cells opened up an avenue for engineering cellular communities based on contact‐dependent communication for different purposes.^[^ ^40^ ^]^ However, a similar contact‐dependent signaling scheme for synthetic cells that utilizes protein–protein interactions at the contact interface between synthetic cells is yet to be realized.

Here, we engineer a light‐based contact‐dependent communication system for synthetic cells. We utilized our previously developed strategy^[^ ^41^ ^]^ for membrane–membrane interface functionalization and repurposed it to engineer a modular contact‐dependent communication system based on intrinsically generated light as a signal. We leveraged optogenetic tools to link the signal, light, to protein recruitment to the membrane interface, supported by mathematical modeling, imitating juxtacrine signaling. The modularity of the design allows for orthogonal contact‐dependent signaling schemes, thus paving the way for engineering complex communication pathways between synthetic cells and between synthetic and natural cells in the future.

## 2 Results

### 2.1 Designing a Contact‐Dependent Light‐Based Communication Mechanism between Synthetic Cells

**Figure 1:**
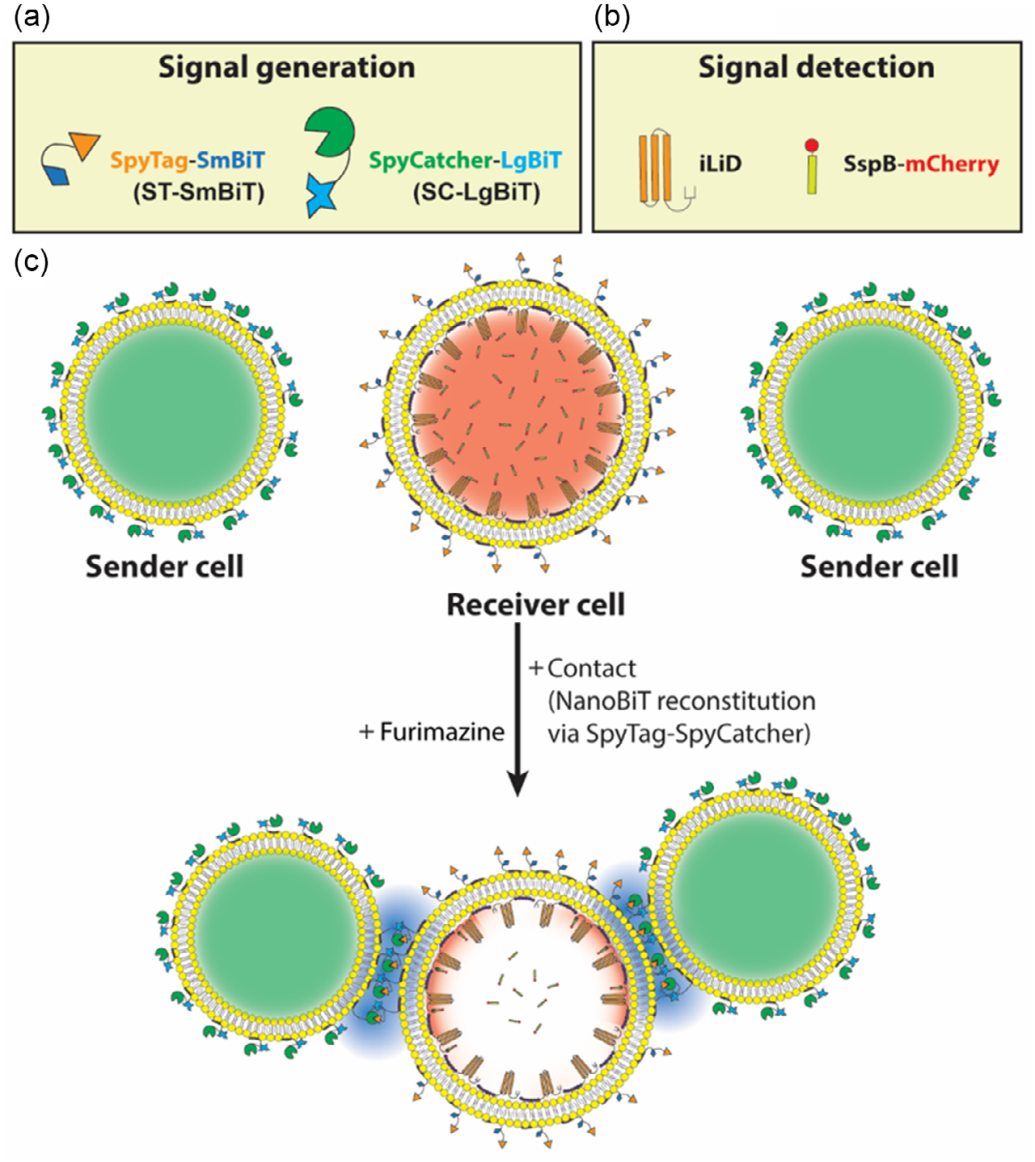
Schematic representation of light‐based synthetic cell communication at synthetic cell membrane‐membrane interfaces. a) Signal generation module consists of ST–SmBiT and SC–LgBiT proteins. SpyTag–SpyCatcher interaction brings the SmBiT and LgBiT fragments close to each other, thereby promoting reconstitution of functional NanoBiT luciferase. b) Signal detection relies on light activation of iLID which induces its dimerization with its binding partner, SspB. SspB is translationally fused to a fluorescent mCherry protein which allows detection of SspB translocation upon signal reception. c) Direct cell–cell contact between sender cells encapsulating green dye and receiver cells encapsulating membrane‐bound iLID and luminal SspB causes SpyTag–SpyCatcher interaction, thus signal generation in the presence of furimazine which in turn promotes iLID activation and translocation of SspB–mCherry from the lumen of receiver cells to the membrane.

Protein modules that send and receive light are crucial building blocks of a light‐based signaling mechanism. In addition, for the signaling cascade to be contact dependent, such protein modules need to be activated and trigger signaling only when synthetic cells are in contact with each other (see **Figure** **1** for further details). We utilized giant unilamellar vesicles (GUVs) as model synthetic cells and sought out appropriate proteins with certain properties that satisfy all aforementioned conditions.

Bioluminescent proteins such as *Nanoluc* (NLuc), *Gaussia* (GLuc), and *Renilla* (RLuc) luciferases are ideal candidates for generating blue light through chemiluminescence reactions. The blue light emitted by these proteins can then activate photosensitive elements such as light–oxygen–voltage sensing (LOV) domains that are present in a variety of different photoactivatable proteins widely used for optogenetics applications.^[^ ^42^ ^]^ Recently, light‐emitting synthetic cells encapsulating GLuc that could activate soil fungi *Trichoderma atroviride* were engineered by Adir et al.^[^ ^35^ ^]^ Additionally, Adir et al. demonstrated photoactivation of the light‐sensitive transcriptional element EL222 by GLuc luminescence inside synthetic cells.^[^ ^35^ ^]^ Thus, the previous successful utilization of luciferases in synthetic cell development made them a prime choice as signal‐generating molecules. Among bioluminescent proteins, NLuc stood out due to its brightness and higher stability compared to its counterparts.^[^ ^43^ ^]^ NLuc catalyzes the oxidation of its substrate, furimazine, in an ATP‐independent manner which results in blue light emission at 450 nm.

Additionally, we wanted the signal generation to only occur when the synthetic cells are in contact with each other. While NLuc had desirable luminescence properties for our design, its function would not be restricted to synthetic cell–cell contact interfaces. Inspired by our previous work on developing InterSpy^[^ ^41^ ^]^ as a strategy for programmable activation of a split fluorescent protein in membrane–membrane interfaces, we reasoned that a split luminescent protein that is activated only at synthetic cell–cell interfaces would be perfectly suited for our design requirements.

A split version of NLuc called Nanoluc Binary Technology (NanoBiT) was designed for reporting protein–protein interactions by Dixon et al.^[^ ^44^ ^]^ NanoBiT consists of a large protein composed of the first nine β sheets of NLuc named large bit (LgBiT) and a small peptide called small bit (SmBiT) that contains NLuc's 10th β‐sheet strand. LgBiT and SmBiT are designed such that they do not have inherent luminescent activity in the presence of furimazine and have low affinity for each other. However, LgBiT and SmBiT complementation and luminescence activation occur when the two fragments are brought together *via* protein–protein interactions. We rationalized that by artificially creating some form of protein–protein interactions between the NanoBiT components exclusively at membrane–membrane interfaces, luminescence activity can be reconstituted in synthetic cell–cell contact interfaces. Since InterSpy relies on SpyTag–SpyCatcher interaction for complementation of a split fluorescent cherry protein (sfCherry) in membrane–membrane interfaces,^[^ ^41^ ^]^ it stood out as an ideal tool for creating the protein–protein interaction required for NanoBiT reconstitution at contact interfaces between sender and receiver synthetic cells. We hypothesized that replacing small and large fragments of sfCherry with SmBiT and LgBiT, respectively, will lead to NanoBiT reconstitution through SpyTag–SpyCatcher interaction exclusively at synthetic cell–cell interfaces. We called the protein made by the fusion of SpyTag and SmBiT as ST–SmBiT and SpyCatcher and LgBiT as SC–LgBiT (Figure 1a).

For signal reception, we selected an improved light‐induced dimerizing protein (iLID) for its compatibility with NLuc luminescence wavelength, high sensitivity, rapid kinetics, and high affinity for its binding partner, stringent starvation protein B (SspB).^[^ ^45^ ^]^ iLID is a relatively small protein that is made from the LOV2 domain from *Avena sativa* (AsLOV2) with seven residues of *Escherichia coli* SsrA peptide at its C terminus. The SsrA strand is sterically inaccessible to its dimerizing partner SspB in the dark. However, upon irradiation of blue light, the AsLOV2 domain undergoes structural rearrangement that releases the SsrA peptide, leading to iLID–SspB dimerization.^[^ ^45^ ^]^ Chakraborty et al. used iLID and SspB Nano as adherent molecules between synthetic cell communities to demonstrate light‐activated communication between synthetic cells.^[^ ^32^, ^36^ ^]^ In their work, one group of synthetic cells encapsulated RLuc to emit light, thereby activating iLID–SspB dimerization and adhesion between synthetic cells. Similarly, iLID–SspB Nano binding on the outer membrane of synthetic cells was demonstrated by Adir et al.^[^ ^35^ ^]^ where N‐terminal fusion of GLuc to iLID made iLID activation through GLuc luminescence possible. These studies provided a strong motivation to exploit iLID as the signal receptor in our design (Figure 1b).

Lastly, we designed the light‐based signaling system such that it resulted in a change in receiver synthetic cells that can be visualized using fluorescence microscopy. In our design, receiver synthetic cells encapsulated membrane‐bound iLID and cytosolic SspB Nano–mCherry (referred to as SspB–mCherry hereafter) while the outer membranes of sender and receiver cells were decorated with SC–LgBiT and ST–SmBiT, respectively. Therefore, when a receiver synthetic cell forms a contact interface with a sender synthetic cell, the NanoBiT activation and luminescence through SpyTag–SpyCatcher interaction drives iLID–SspB dimerization, leading to membrane‐recruitment of mCherry (Figure 1c), which can be detected *via* fluorescence microscopy.

### 2.2 SpyTag–SpyCatcher‐Mediated NanoBiT Reconstitution

**Figure 2:**
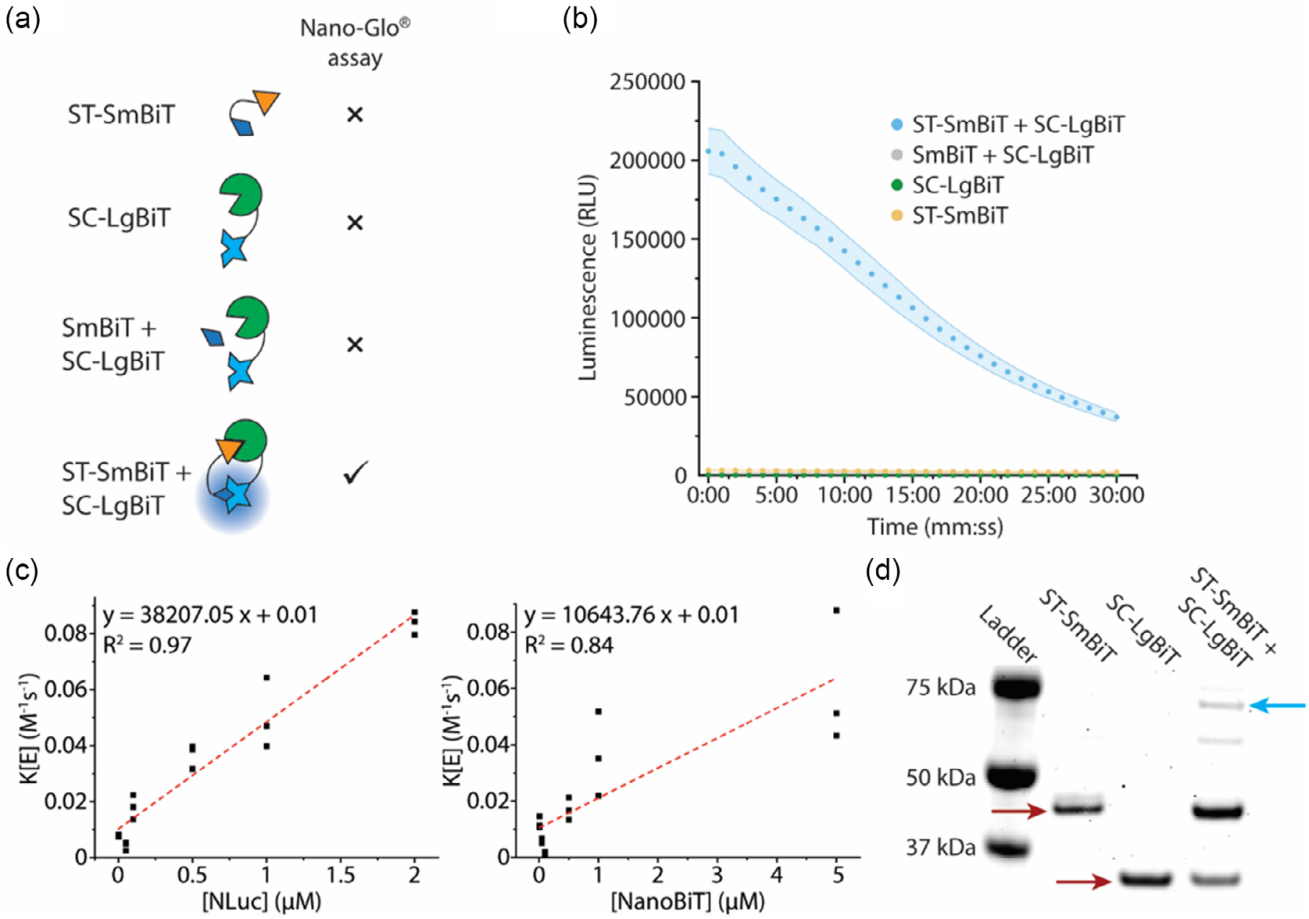
Reconstitution of NanoBIT activity using SpyTag and SpyCatcher. a) Schematic of the luciferase assay based on Promega Nano–Glo assay. The left column illustrates different combinations of proteins while the right column represents expected readouts from Nano–Glo assay. Functional reconstitution of NanoBiT only occurs in the presence of protein–protein interaction induced by SpyTag–SpyCatcher dimerization. b) The luminescence readout from four different reactions represented in (a). Data is represented as mean ± S.D., *n* ≥ 3. c) Regression analysis on the relationship between K[E], representing the slope of log(photon flux) versus time plot, and NLuc (left) or NanoBiT (right) concentration determines the enzyme rate constant. K is the rate constant, and [E] is enzyme concentration. *n* = 3. d) In‐gel fluorescence imaging of the Coomassie‐stained ladder (lane 1), ST–SmBiT (lane 2, red arrow), SC–LgBiT (lane 3, red arrow), and the mixture of SC–LgBiT and ST–SmBiT forming a dimer (lane 4, cyan arrow).

To test our hypothesis on whether SpyTag–SpyCatcher interaction can lead to functional NanoBiT reconstitution, we made constructs SC–LgBiT and ST–SmBiT by fusing SpyCatcher and SpyTag to the N terminus of LgBiT and C terminus of SmBiT, respectively. Since in our design, SC–LgBiT and ST–SmBiT were eventually on the outer surface of sender and receiver synthetic cells (Figure 1c), we designed the constructs such that SC–LgBiT and ST–SmBiT had a C‐terminal and a N‐terminal 6xHis tag, respectively, so that the molecules could bind to lipids with nitriloacetic acid (NTA)–Ni headgroups. We then asked whether ST–SmBiT, SC–LgBiT, a 1:1 mixture of ST–SmBiT and SC–LgBiT, or a control experiment where SC–LgBiT was mixed with SmBiT, lacking the SpyTag domain, has luminescence activity. To detect luminescence, we utilized Nano–Glo assay (**Figure** **2**a). After we made mixtures of 500 nM of our designed proteins or their combination and incubated them to ensure SpyTag–SpyCatcher bond formation, we observed that only the mixture of ST–SmBiT and SC–LgBiT demonstrated luminescence for about 30 min following furimazine addition (Figure 2b). Notably, the mixture with SC–LgBiT and SmBiT did not show any luminescence signal, highlighting the low affinity of SmBiT and LgBiT and the requirement of SpyTag–SpyCatcher isopeptide bond formation in reconstituting NanoBiT as a functional luciferase.

Using luminescence imaging, we measured the total photon flux from luminescence reactions catalyzed by NLuc or reconstituted NanoBiT to compare light emittance of NanoBiT with its full‐length counterpart, NLuc (Figure S1a,b, Supporting Information). We observed that NLuc consistently had a higher maximum total photon flux over a range of concentrations (Figure S1c, Supporting Information). This is expected as Dixon et al. also showed that lysates of cells transfected with equal amounts of NanoBiT DNA in the presence of rapamycin demonstrated lower luminescence activity compared to lysates with NLuc.^[^ ^44^ ^]^ We also attempted to compare the kinetics of NLuc and NanoBiT catalytic reactions. We correlated the measured total photon flux to the exponential decay of the substrate through its exhaustion by NLuc or NanoBiT. By modeling the luminescence reaction as an ordinary differential equation, we calculated the catalytic rate constants of NLuc and NanoBiT. We found that NLuc showed a higher rate constant than NanoBiT (≈38 207 ± 2450 M^−1^s^−1^ versus ≈10 644 ± 1734 M^−1^s^−1^ [95% confidence interval]) (Figure 2c).

In addition to the luminescence measurements, sodium dodecyl sulfate‐polyacrylamide gel electrophoresis (SDS–PAGE) analysis of SC–LgBiT and ST–SmBiT mixture confirmed dimer formation mediated by SpyTag–SpyCatcher interaction (Figure 2d). We also saw an additional band below the expected band, the reason for which is unclear to us. Expectedly, in the control reaction where SC–LgBiT was mixed with SmBiT, no evidence of dimer formation was found (Figure S2, Supporting Information). Interestingly, we observed monomeric ST–SmBiT and SC–LgBiT in our SDS–PAGE analysis indicating that only a portion of ST–SmBiT and SC–LgBiT molecules formed heterodimers. This observation is aligned with our previous work^[^ ^41^ ^]^ on SpyTag–SpyCatcher‐mediated sfCherry reconstitution in which we noted inefficient dimer formation as well. Additionally, our evidence on inefficient NanoBiT reconstitution provided a possible reason for the lower luminescence of NanoBiT compared to NLuc (Figure S1c, Supporting Information). Nevertheless, since reconstituted NanoBiT demonstrated bright luminescence in bulk and complete dependence on SpyTag–SpyCatcher interaction for its functional reconstitution, we concluded that ST–SmBiT and SC–LgBiT are well suited as building blocks of our contact‐dependent signal generation scheme.

### 2.3 SspB–iLID Dimerization by External Illumination in GUVs

**Figure 3:**
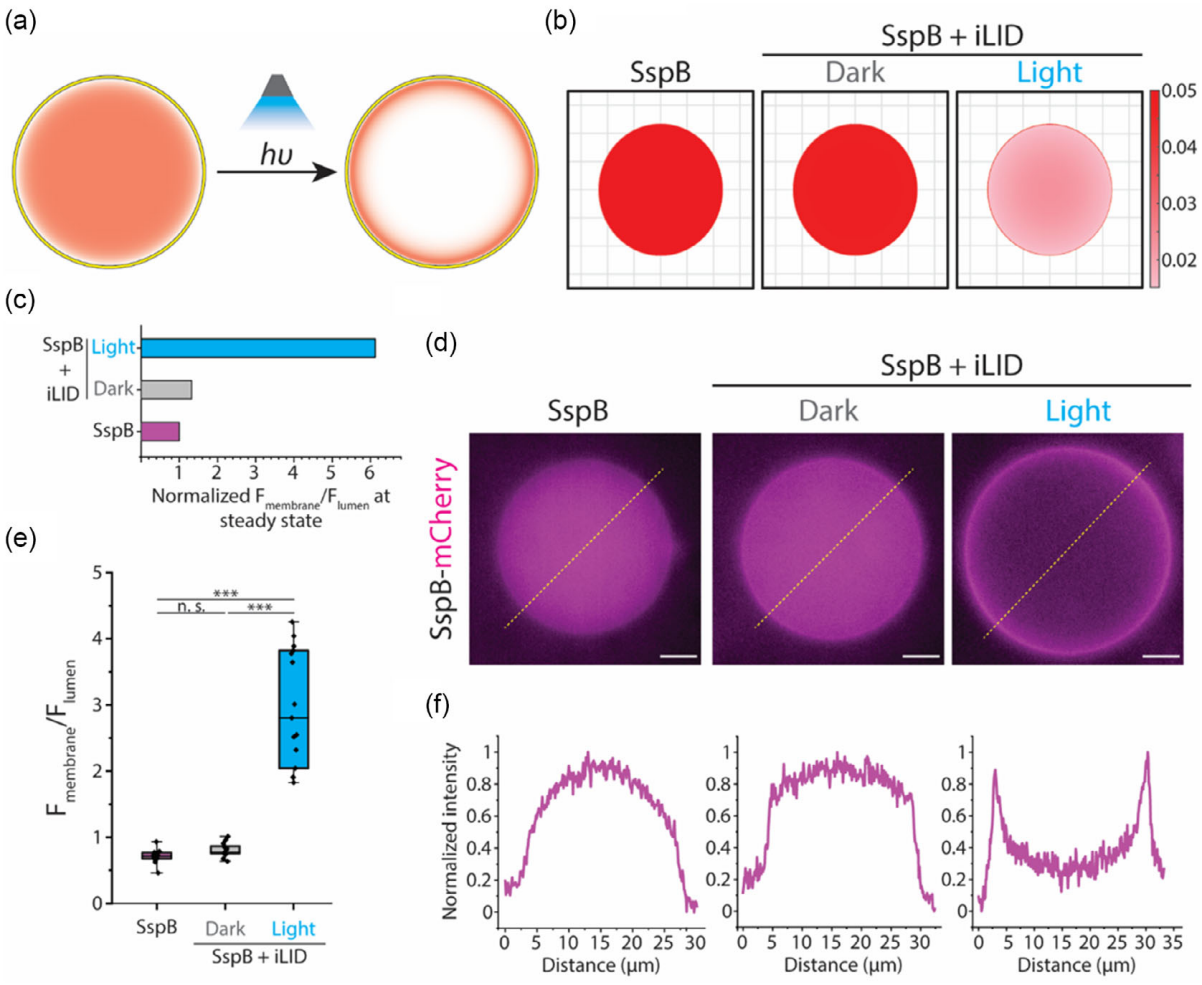
Light illumination drives SspB and iLID interaction inside a GUV. a) Schematic representing iLID and SspB encapsulated inside a GUV. SspB–iLID dimerization is induced by external blue light. SspB is illustrated with red color in the lumen of GUV. The depiction of membrane‐bound iLID is omitted for simplicity. b) Representative results of the mathematical model calculating steady‐state SspB concentration inside a 20 μm GUV either encapsulated alone or co‐encapsulated with membrane‐bound iLID and exposed to dark or light. c) Bar graph depicting the normalized ratio of SspB–iLID to luminal SspB at the steady‐state calculated based on the mathematical model. d) Representative fluorescence images of SspB–mCherry encapsulated in GUV (left), SspB–mCherry co‐encapsulated in GUV with membrane‐bound iLID in the dark (middle), and SspB–mCherry co‐encapsulated in GUV with membrane‐bound iLID and exposed to external blue light for 15 min (right). Scale bars: 5 μm. e) Boxplots comparing the ratio of SspB‐mCherry fluorescence intensity at the GUV membrane to the luminal fluorescence intensity in GUVs encapsulating SspB–mCherry, co‐encapsulating SspB–mCherry and iLID in the dark, or co‐encapsulating SspB–mCherry and iLID exposed to blue light. The data shows the average ratio of SspB–mCherry signal at the membrane of the GUV to the luminal SspB–mCherry signal with background subtraction for 30 points for 15 different GUVs. The box represents the 25–75th percentiles, and the median is indicated. The whiskers show the minimum and maximum data points, *n* = 15 from four independent experiments. f) Intensity profiles of the SspB–mCherry along the dashed yellow line in (d). Each plot corresponds to the image in (d). *p* values are calculated using one‐way ANOVA test and corrected using Tukey's honest significant difference. n.s. denotes not significant and *** represents *p* < 0.001.

Once we determined the signal‐generation elements in our signaling cascade, we sought to assess the functionality of our signal reception molecule, iLID. We started by asking whether membrane‐bound iLID inside GUVs can be activated by external blue light illumination. In the presence of cytosolic SspB–mCherry, we expected to observe SspB–mCherry recruitment to the GUV membrane through SspB–iLID dimerization upon blue light irradiation (**Figure** **3**a).

We used a theoretical approach to evaluate whether the amount of SspB–mCherry recruitment to the GUV membrane is enough such that it allows detection of SspB–mCherry at the membrane using fluorescence microscopy. We simulated SspB–mCherry membrane translocation by modeling the SspB–iLID dimerization reaction as well as SspB–mCherry diffusion inside GUVs. Based on the work of Guntas et al. on iLID development, we chose association constants for iLID–SspB dimerization that reflect their high affinity under the blue light illumination (see Methods section, Supporting Information).^[^ ^45^ ^]^ Using a finite difference method, we simulated SspB–iLID dimerization both in dark and under blue light stimulation and computed the steady‐state location of SspB–mCherry inside a 20 μm diameter circle (Figure 3b).

Our modeling clearly showed that SspB–mCherry recruitment to the membrane occurs only in the presence of both iLID and light illumination (Figure 3b, right). Additionally, the ratio of SspB molecules at the membrane to cytosolic SspB molecules in the presence of light was estimated to be around 6 (Figure 3c). This ratio would allow us to visualize SspB–mCherry recruitment to the membrane *via* fluorescence microscopy. To test this, we generated GUVs with phospholipid membranes made of POPC and 5% DGS–NTA (Ni). POPC vesicles were reported to have superior light transmission properties compared to other phospholipid molecules commonly used in synthetic cells.^[^ ^35^ ^]^

We encapsulated 450 nM iLID and 100 nM SspB–mCherry inside GUVs and used fluorescence microscopy to detect SspB–mCherry. While SspB–mCherry remained in the lumen of the GUV, iLID was recruited to the membrane due to its C‐terminal polyhistidine–tag affinity to NTA (Ni) group on DGS–NTA (Ni) lipids in the GUV membrane. Using the aforementioned concentrations for iLID and SspB along with considering the size, lipid concentration, and lipid composition of the GUVs ensures neither NTA (Ni) nor iLID saturation occurs in our experiments (see Section S6, Supporting Information, for details). Thus, the effect of GUV size variation on the ratio of membrane to lumen signal is negligible. When the GUVs were exposed to external blue light illumination for 15 min, we observed strong membrane translocation of SspB–mCherry only in external blue‐light‐exposed GUVs that encapsulated membrane‐bound iLID (Figure 3d). To confirm iLID localization to the membrane was due to the His–tag–NTA (Ni) affinity, we tested light‐dependent SspB–mCherry membrane recruitment using a fluorescein isothiocyanate (FITC)‐labeled iLID. Our results indicated that while iLID–FITC is membrane‐bound regardless of external light irradiation, SspB–mCherry is localized to the membrane only in the presence of light stimulation (Figure S3, Supporting Information).

Our quantification of SspB–mCherry inside GUVs showed that the mCherry intensity at the membrane of GUVs encapsulating iLID and exposed to blue light was on average three times higher than luminal SspB–mCherry intensity (Figure 3e). This ratio is half of what our model predicted, and the discrepancy can be attributed to the 3D effects that are present only in experimental system. In addition, it has been shown that iLID switching is less efficient when iLID is linked to the membrane.^[^ ^46^ ^]^ This phenomenon is absent in the modeling and can also explain the difference in the experimental and modeling results.

Importantly, iLID–SspB–mCherry dimerization revealed itself in the fluorescence intensity profile of SspB–mCherry along the diameter of the GUV as clear peaks in signal intensity at the membrane that is present only for stimulated GUVs encapsulating both iLID and SspB–mCherry (Figure 3f, right). A sudden increase in signal intensity along the GUV diameter can also be noticed in non‐stimulated GUVs containing iLID and SspB–mCherry, which suggests some iLID–SspB–mCherry dimerization in the dark (Figure 3f, middle). In contrast, GUVs encapsulating only SspB–mCherry did not exhibit a peak nor such a sudden increase in their fluorescence intensity profile analysis (Figure 3f, left). Taken together, our results indicate that in our synthetic cell system, membrane‐bound iLID can be photoactivated by external blue light as demonstrated through cytosolic SspB–mCherry translocation to the synthetic cell membrane.

### 2.4 NLuc‐Mediated SspB–iLID Dimerization

While iLID–SspB dimerization was successfully driven by external light illumination, in our designed contact‐dependent signal transduction scheme, iLID excitation was required to occur through a luminescence signal from a reconstituted luciferase (Figure 1c). Therefore, we set out to test if iLID can be activated by luminescence in synthetic cells. To generate luminescence, we used NLuc as the luciferase due to its bright and sustained signal. Photoactivation of LOV domain by various luciferases including NLuc has been shown both in natural and synthetic cells. While Kim et al. demonstrated that LOV activation by NLuc through bioluminescence resonance energy transfer required NLuc and LOV proximity on the same side of the membrane (*cis* activation),^[^ ^47^ ^]^ Chakraborty et al. reported that encapsulated RLuc could activate iLID even when the two molecules reside on different sides of the membrane (*trans*‐activation).^[^ ^32^ ^]^ Since the signal from luciferase must cross the membrane to activate the iLID in our design, we nex*t* tested if NLuc can successfully induce SspB–mCherry membrane translocation when it is either inside or outside of the GUV.

**Figure 4:**
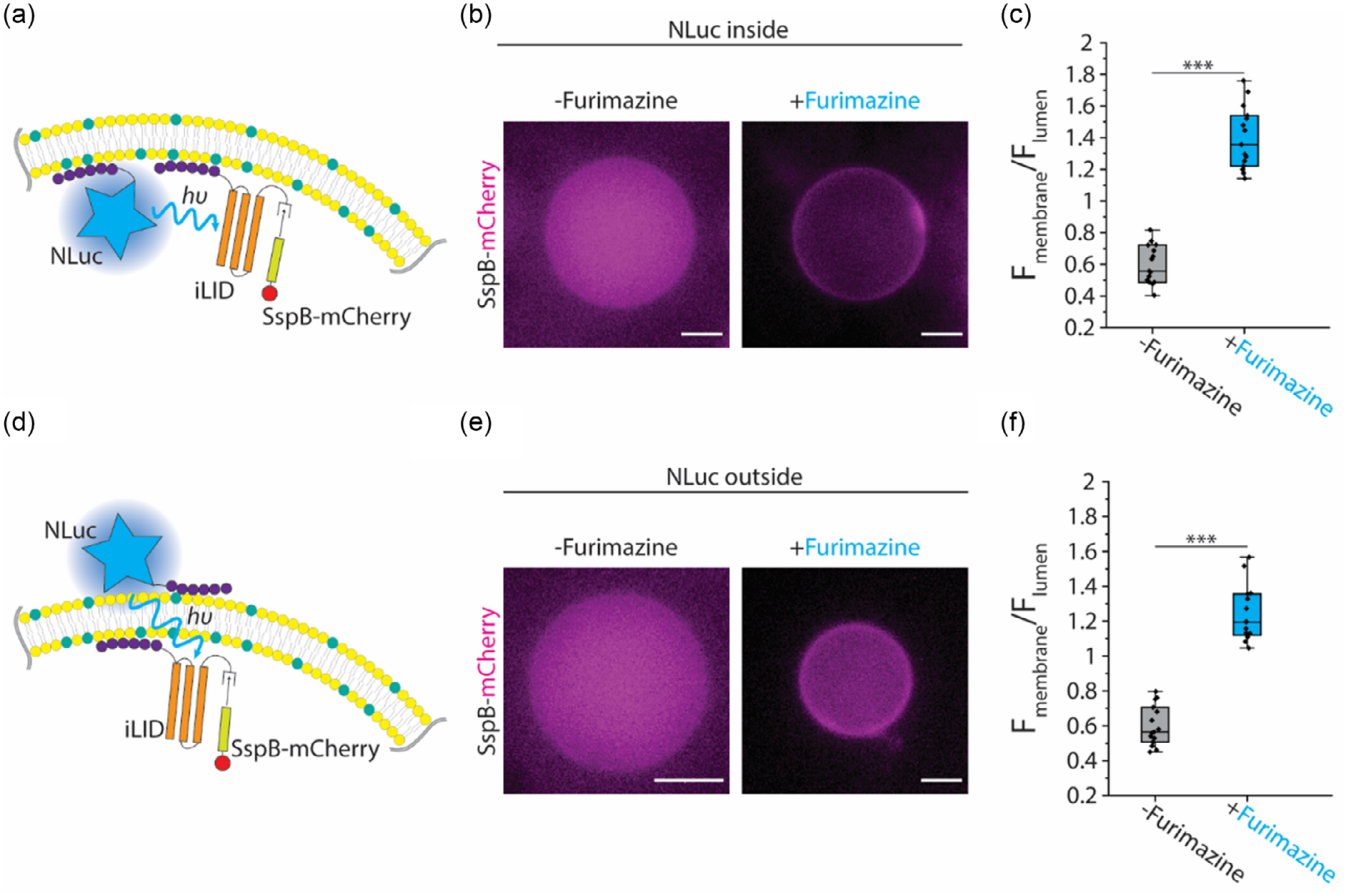
Membrane‐bound NLuc drives iLID and SspB interaction. Schematic illustrating membrane‐bound iLID activation by NLuc linked to a) the inner or d) outer membrane of GUV that drives SspB–mCherry dimerization with iLID. Representative fluorescence images of SspB–mCherry co‐encapsulated with iLID and NLuc in a GUV with NLuc b) inside or e) outside in the absence (left) or presence (right) of NLuc substrate, furimazine. Scale bars: 5 μm. Boxplots comparing the ratio of SspB–mCherry fluorescence intensity at the membrane of GUV to the luminal SspB–mCherry fluorescence intensity with NLuc c) inside or f) outside in the absence or presence of furimazine. The data shows the average ratio of SspB–mCherry signal at the membrane of the GUV to the luminal SspB–mCherry signal with background subtraction for 30 points for 15 different GUVs. The box represents the 25–75th percentiles, and the median is indicated. The whiskers show the minimum and maximum data points, *n* = 15 from 4 independent experiments. *p* values are calculated using two‐tailed Welch's *t*‐test. *** represents *p* < 0.001.

First, we purified NLuc with a C‐terminal polyhistidine tag and confirmed its peak luminescence around 450 nm (Figure S4, Supporting Information) which is compatible with iLID excitation wavelength.^[^ ^45^ ^]^ Next, we encapsulated NLuc, iLID, and SspB–mCherry in GUVs with membranes composed of POPC and DGS–NTA (Ni), allowing binding of NLuc and iLID to the membrane due to the polyhistidine tag and NTA (Ni) affinity. We hypothesized that the close proximity between NLuc and iLID on the membrane would lead to photoactivation of iLID by NLuc luminescence and subsequent SspB–mCherry membrane translocation (**Figure** **4**a). Consistent with our expectation, we observed that upon addition of furimazine, which is a membrane permeable substrate, SspB–mCherry was recruited to the membrane (Figure 4b). Interestingly, the extent of SspB–mCherry membrane translocation was lower when iLID was excited by intracellular NLuc (Figure 4c) compared to its excitation by external light (Figure 3e). While SspB–iLID dimerization is fast, the time required for ample, detectable SspB–mCherry membrane recruitment is limited by the diffusion of free, luminal SspB–mCherry molecules. Therefore, achieving high contrast between membrane‐localized and luminal SspB–mCherry would take longer for larger GUVs. Since our measurements demonstrated that luminescence of NLuc and NanoBiT decay overtime, in the experiments where iLID was activated by NLuc or NanoBiT, we fed the GUV solution with fresh furimazine every 15 min to maintain light generation, thereby inducing more detectable SspB–mCherry membrane localization.

Upon confirming iLID *cis*‐activation, we sought to see whether *trans*‐activation of iLID by NLuc is possible in our synthetic cell system (Figure 4d) because iLID excitation by NanoBiT occurs through *trans*‐activation in our signal transduction design. We encapsulated iLID and SspB–mCherry in POPC GUVs doped with DGS–NTA (Ni) and mixed the GUVs with 500 nM of His‐tagged NLuc and incubated the mixture to ensure binding of NLuc to the GUV outer membrane. Then, we washed the GUV mixture to remove unbound NLuc from the outer solution. Upon addition of furimazine, we observed SspB–mCherry recruitment to the membrane (Figure 4e), indicating *trans*‐activation of iLID by NLuc linked to the outer membrane of a GUV. Our control experiments confirmed that the SspB–mCherry translocation was indeed induced by the membrane‐bound NLuc as exclusion of either NLuc or NTA (Ni) lipids caused no SspB–mCherry membrane recruitment (Figure S5a,b, Supporting Information). We note that similar to when NLuc was encapsulated inside GUVs, the extent of NLuc‐mediated iLID activation was found to be significantly lower than activation by external light (Figure 4f and Figure S6, Supporting Information). The weaker activation of iLID by NLuc in comparison with external light could be caused by both the lower number of photons emitted by NLuc as well as the constant decay of NLuc photons due to substrate exhaustion.

Further, luminescence measurements of NLuc bound to either inside or outside of GUVs revealed different kinetics of NLuc luminescence (Figure S7, Supporting Information). We measured an amplified signal from NLuc linked to the outer membrane of GUVs compared to encapsulated NLuc. In contrast, encapsulated NLuc demonstrated prolonged signal generation. The difference in the amplitude and kinetics of luminescence was due to the fact that 500 nM NLuc in the GUV inner solution contains fewer NLuc molecules compared to the outer solution. In contrast, the concentration of added substrate in both cases was the same. Therefore, the prolonged luminescence of encapsulated NLuc was caused by excessive substrate present in the outer solution. Taken together, our results demonstrate the ability of NLuc to activate iLID by both *cis‐* and *trans*‐activation mechanisms. Thus, we hypothesized that replacing NLuc with NanoBiT would similarly lead to iLID activation, thereby realizing our designed contact‐dependent light‐based signaling mechanism.

### 2.5 Contact‐Dependent Light‐Based Synthetic Cell Communication

Since in our design (Figure 1c) the juxtacrine signaling occurs through the interaction of SpyTag and SpyCatcher driving NanoBiT reconstitution, we next asked whether NanoBiT luminescence can induce iLID activation akin to NLuc‐mediated signaling (Figure 4d). To test this, we generated receiver synthetic cells encapsulating membrane‐bound iLID and luminal SspB–mCherry that displayed ST–SmBiT on their outer membrane. To induce NanoBiT formation, we introduced the complementary fragment of NanoBiT, SC–LgBiT, attached to small unilamellar vesicles (SUVs) as an extracellular “signal” (as depicted in Figure S8a, Supporting Information). Utilizing this approach enables NanoBiT reconstitution at the GUV–SUV membrane–membrane interfaces at a high rate, thus allowing us to measure NanoBiT activation and luminescence through detecting SspB–mCherry membrane recruitment.

After mixing SUV‐bound SC–LgBiT with the GUVs harboring ST–SmBiT, we measured a strong luminescence signal upon addition of furimazine, indicating successful NanoBiT reconstitution at the SUV–GUV interface (Figure S8b, Supporting Information). In alignment with our bulk measurements (Figure 2b), receiver cells harboring SmBiT lacking the SpyTag domain showed no luminescence (Figure S8b, Supporting Information).

Further, we tested if NanoBiT luminescence can induce iLID activation and subsequent SspB–mCherry membrane translocation. Our results indicated SspB–mCherry recruitment to the membrane in the presence of both furimazine and ST–SmBiT on the GUVs (Figure S8c–e, Supporting Information, for a cohort of receiver cells before and after addition of furimazine). Additionally, we monitored SspB–mCherry membrane translocation upon addition of furimazine over time. We found that SspB–mCherry membrane recruitment reached its maximum state around 10 min after the addition of furimazine and maintained a similar membrane‐proximal signal at 15 min, before slowly returning to the GUV lumen over the next 40 min (Figure S8f, Supporting Information). This long period of SspB–mCherry dissociation from membrane is likely due to the previously reported slow reversal kinetics of iLID–SspB Nano binding.^[^ ^48^, ^49^, ^50^ ^]^ These results collectively demonstrate that NanoBiT complementation on the surface of receiver cells upon contact with membrane‐bound SC–LgBiT molecules reconstitutes a functional luciferase that drives iLID–SspB dimerization.

Ultimately, our characterization of individual modules for signal generation and reception along with the design of NanoBiT‐mediated signal transduction allows the demonstration of contact‐dependent light‐based communication among synthetic cells. To do so, we generated two distinct populations of synthetic cells made by POPC and a small amount of DGS–NTA (Ni). Sender cells encapsulated FITC, a green fluorescence dye, for identification purposes and harbored SC–LgBiT on their outer membrane. Receiver cells, in contrast, were decorated with ST–SmBiT on their outer membrane while encapsulating membrane‐bound iLID and luminal SspB–mCherry.

**Figure 5:**
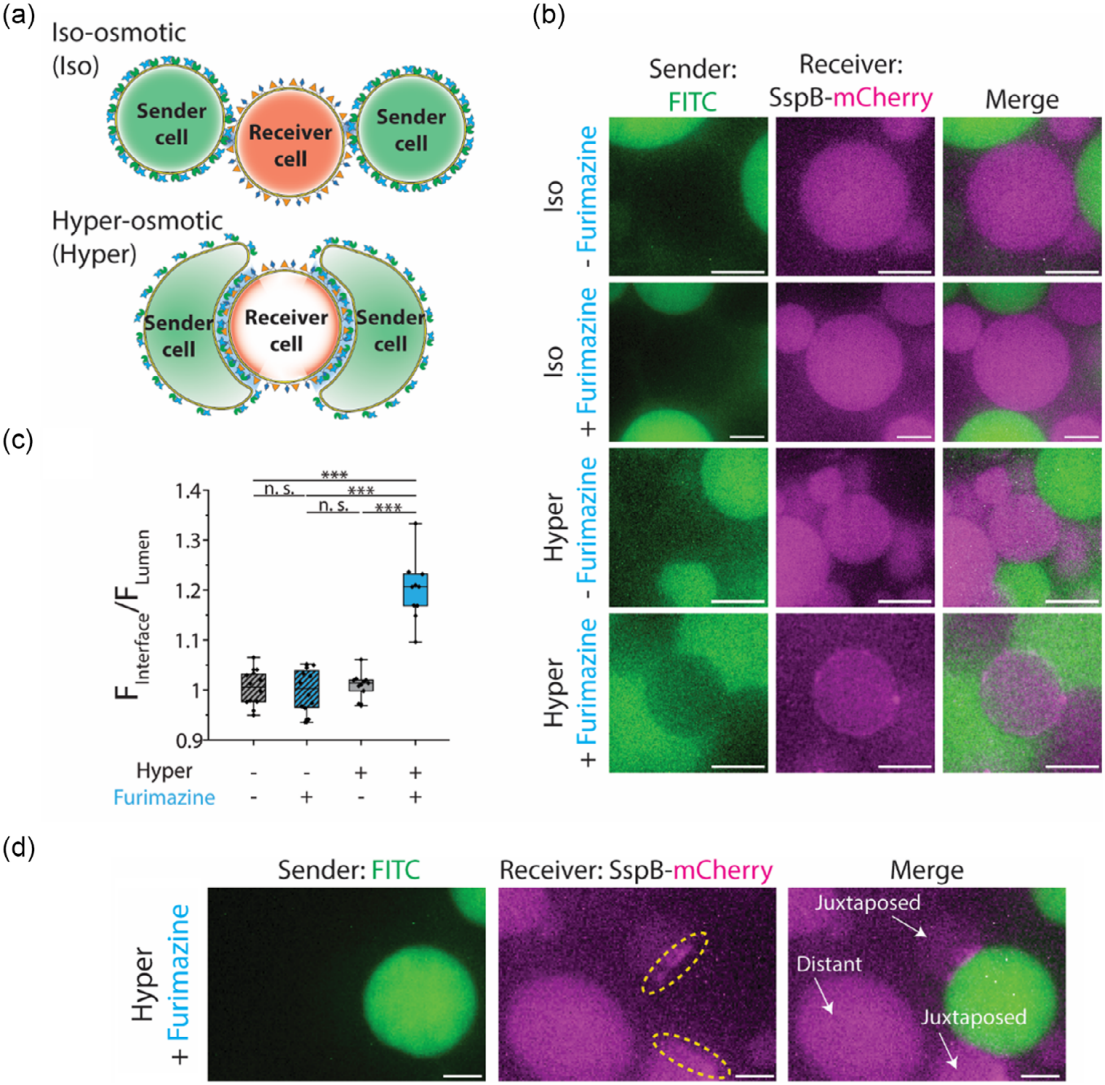
Contact‐dependent light‐based communication between synthetic cells. a) Schematic of light‐based contact‐dependent communication between synthetic cells. Top: in the iso‐osmotic condition, the small contact area between sender and receiver cells depicted in green and red, respectively, does not result in sufficient SpyTag–SpyCatcher‐mediated NanoBiT reconstitution to induce detectable dimerization of SspB–mCherry with iLID. Bottom: in the hyperosmotic, deflated GUVs form large interfaces that cause sufficient SpyTag–SpyCatcher‐assisted NanoBiT reconstitution. Functional reconstitution of NanoBiT then activates membrane‐bound iLID inside receiver cells that leads to SspB–mCherry recruitment to the receiver cell inner membrane. SspB–mCherry is illustrated in red and the depiction of iLID is omitted for simplicity. b) Representative confocal images of FITC (green) encapsulated in sender cells and SspB–mCherry (magenta) encapsulated in receiver cells. iLID activation and SspB–mCherry membrane localization only occur in the hyperosmotic condition and in the presence of furimazine. Scale bars: 10 μm. c) Box plots comparing the ratio of the SspB–mCherry fluorescence intensity at the GUV membrane over the luminal SspB–mCherry fluorescence intensity in the presence or absence of hyperosmotic condition and furimazine. The data shows the average ratio of SspB–mCherry signal to the luminal SspB–mCherry signal for membrane–membrane interfaces of sender and receiver cells for at least 10 different receiver cells. The box represents the 25–75th percentiles, and the median is indicated. The whiskers show the minimum and maximum data points, *n* ≥ 10 from at least 4 independent experiments. *p* values are calculated using two‐way ANOVA test and corrected using Tukey's honest significant difference. n.s. denotes not significant and *** represents *p* < 0.001. d) Representative confocal image of a sender cell encapsulating FITC (green) and receiver cells encapsulating SspB–mCherry (magenta). Juxtaposed receiver cells display SspB–mCherry translocation to the sender cell–receiver cell interface while SspB–mCherry remains luminal in distant receiver cell. Scale bars: 5 μm.

While SpyTag–SpyCatcher interaction brings the NanoBiT fragments close and promotes formation of the functional luciferase in solution, we found that it was unable to adhere neighboring sender and receiver cells for detectable SspB–mCherry membrane recruitment in the presence of furimazine. To circumvent this challenge, we resorted to using hyperosmotic condition to make GUVs deflated and deformable.^[^ ^51^, ^52^ ^]^ We rationalized that in a hyperosmotic solution, a strategy also used by others to generate synthetic cell contacts,^[^ ^36^, ^52^ ^]^ deflated synthetic cells will form large interfaces with ample NanoBiT reconstitution, thereby allowing visualization of SspB–mCherry membrane translocation (**Figure** **5**a).

We mixed equal amounts of sender and receiver cells in the presence or absence of both hyperosmotic condition and furimazine and probed whether SspB–mCherry was recruited to the membrane. The contact interfaces between sender and receiver cells remained stable throughout our experiments. Confocal fluorescence microscopy images of receiver synthetic cells revealed SspB–mCherry membrane translocation only in the case where furimazine was added to the mixture of synthetic cells in the hyperosmotic condition (Figure 5b). We note that these were not the same sender–receiver cells before and after furimazine addition. Analyzing the SspB–mCherry fluorescence along the interfaces of multiple sender–receiver cell pairs further verified the significant increase in the amount of SspB–mCherry at the membrane of receiver cells that were exposed to both hyperosmotic condition and furimazine (Figure 5c). Additionally, we observed that only juxtaposed receiver cells had SspB–mCherry translocation to the interface while SspB–mCherry remained luminal in distant receiver cells (Figure 5d). In contrast, our control experiment with receiver cells harboring SmBiT, lacking SpyTag domain, did not lead to any change in SspB–mCherry localization, confirming that the SspB–mCherry membrane translocation is due to the NanoBiT reconstitution mediated by SpyTag–SpyCatcher interaction (Figure S9a,b, Supporting Information). Taken together, our results realize a molecular platform for contact‐dependent light‐based signaling between synthetic cells.

## 3 Discussion

Contact‐dependent signaling and communication evolved to enable precise targeting and highly specific communication between cells, making essential processes such as differentiation and phagocytosis possible. With the increasing interest toward synthetic multicellular systems,^[^ ^53^ ^]^ development of a synthetic contact‐dependent communication tool promises biomimicry of various biologically relevant functionalities in synthetic tissues that unlocks synthetic cell applications in regenerative medicine and drug delivery. We demonstrated the design and implementation of a protein‐based light‐activated contact‐dependent communication system that enables contact‐dependent signaling between synthetic cells. We developed the signal generation and detection elements of our signaling scheme such that it enabled signaling through light *via* split proteins reconstituted exclusively at the synthetic cell interfaces. Previously demonstrated light‐based signaling utilized light as an intermediary means to trigger chemical communication^[^ ^31^, ^32^ ^]^ while here we demonstrated using light as the primary signal for communication between synthetic cells.

To make the signaling dependent on physical contact between synthetic cells, we selected a split luciferase protein, NanoBiT, as a signal‐generating module and made its function contingent on protein–protein interaction. We leveraged SpyTag–SpyCatcher interaction to permanently bring together the two fragments of NanoBiT and demonstrated SpyTag–SpyCatcher‐mediated functional reconstitution of NanoBiT. Inside receiver cells, we encapsulated a light‐switchable protein, iLID, and showed its activation triggered by signals coming either from an external light source or a membrane‐bound luciferase protein. Lastly, we combined signal generation and reception modules and demonstrated NanoBiT reconstitution in the sender and receiver synthetic cell interfaces which led to a molecular translocation event in receiver cells.

Importantly, NanoBiT reconstitution at the surface of a receiver cell as shown in Figure S8, Supporting Information, can act as a signal transduction machinery where the signal (i.e., light) is transduced across the membrane of a receiver cell possessing a “receptor” unit (ST–SmBiT) once it recognizes a signal (SC–LgBiT–SUV). This mechanism holds potential as a platform for synthetic signal transduction as, unlike many naturally occurring receptor proteins, it does not require a membrane protein to facilitate signaling crossing the membrane. However, it should be noted that compared to GUVs, since SUVs are much smaller, their addition to receiver GUVs in a large concentration would lead to the complete decoration of receiver GUV membranes, thus activating the response mechanism uniformly across the GUV membrane (as shown in Figure S8, Supporting Information). In contrast, a GUV sender cell would form a limited interface with a receiver cell, thus limiting the spatial extents of response generation within the receiver cell (as shown in Figure 5d).

Limited by the simplicity of GUVs as our model synthetic cells, we relied on the hyperosmotic condition to induce formation of large interfaces between synthetic cells. An avenue worth exploring in the future to improve our design is decorating the GUV membrane with adhesion molecules such as E‐cadherin or claudin^[^ ^54^ ^]^ to mimic cell adhesion in synthetic cell communities. In addition, we reported that compared to excitation by external light, NLuc‐ or NanoBiT‐mediated iLID activation was attenuated (Figure S6, Supporting Information). This is possibly linked to inefficient energy transfer through radiative and non‐radiative means and incomplete NanoBiT reconstitution (Figure 2d and Figure S2, Supporting Information). Furthermore, while SpyTag–SpyCatcher bond formation is relatively quick (less than 10 min), the split protein reconstitution and maturation are slower.^[^ ^41^ ^]^ Thus, we waited 30 min for membrane interface formation and began monitoring SspB translocation 15 min after substrate addition. To circumvent inefficient signaling, one can imagine signaling amplification schemes in which a light‐sensitive enzyme, such as a light‐sensitive kinase,^[^ ^55^ ^]^ is activated by light and continues the signaling cascade by phosphorylation of downstream targets. An alternative approach is to explore different dimerizing protein pairs like coiled‐coil domains to potentially increase or even tune (i.e., using engineered coiled‐coil forming peptides^[^ ^56^ ^]^) the efficiency of NanoBiT reconstitution. Lastly, further quantitative characterization of iLID activation by luminescence sources in the future would enable a more systematic understanding of light‐based signaling, thereby paving the way for more efficient designs.

Another area of future improvement for our study would be developing a platform for consistent GUV–GUV interface formation. In our work and similar studies,^[^ ^32^, ^33^, ^36^ ^]^ generation of sender cell–receiver cell contact interfaces depends on GUV mixing and random positioning. Using microfluidic trap devices^[^ ^57^ ^]^ or single‐GUV manipulation tools such as optical tweezers^[^ ^52^ ^]^ would potentially circumvent this challenge. In addition, our juxtacrine signaling platform can potentially be switched on multiple times by periodic introduction of furimazine to the synthetic cell solution. However, in our system, substrate exhaustion is the primary means of stopping communication. While the NanoBiT reconstitution is irreversible in this work due to the nature of SpyTag–SpyCatcher interaction, replacing the SpyTag/SpyCatcher pair with other non‐covalently dimerizing proteins with high affinity such as specific synthetic zipper proteins^[^ ^58^ ^]^ can lead to a more dynamic and tunable signaling design.

Previously, we engineered a protein‐based tool called InterSpy for the reconstitution of a split fluorescent protein at the membrane–membrane interface between natural and synthetic cells. Here, we extended InterSpy to accommodate signaling between synthetic cells. We chose light as the signal as it does not require auxiliary proteins such as channels and transporters to cross the lipid membrane. Further, the expansive repertoire of optogenetics proteins allows modular designs of the signaling cascade, thus widening the applications. In practice, our designed signaling cascade is analogous to an AND gate which requires physical contact and furimazine to generate light as its output.

While we showed light activation of iLID and SspB membrane recruitment as the response to signal in receiving cells, more complicated responses based on transcription–translation can be engineered using this AND gate by switching iLID with appropriate optogenetics proteins. For instance, using light‐activated transcription elements such as light‐sensitive T7,^[^ ^59^ ^]^ bacterial light‐inducible transcription factor EL222,^[^ ^60^ ^]^ or a light‐sensitive Cre recombinase,^[^ ^61^, ^62^ ^]^ one can recapitulate contact‐dependent induction of gene expression, a process that resembles Notch–Delta signaling, the practical demonstration of which awaits further studies. Alternatively, lipid‐modifying enzymes, such as phosphoinositide (PI) kinases or phosphatases, can be recruited to the contact site to regulate PI synthesis and degradation which is crucial in cellular signal transduction in natural biological systems.^[^ ^63^ ^]^ Contact‐dependent signal generation can also be used to reconstruct a “synthetic synapse” in the future by coupling it with a light‐gated ion channel such as channelrhodopsin to allow electrical excitation of receiver cells in the presence of furimazine. Finally, one can envision light‐based juxtacrine signaling to drive actin or microtubule assembly at the contact sites using light‐activated motor proteins^[^ ^64^ ^]^ to regulate receiver cell mechanics, a feature that resembles signaling events in natural immunological synapses.

A foundational element of our signaling design was utilizing NanoBiT for signal generation. A split protein that requires protein–protein interaction for its function allowed us to make light generation conditional on physical contact between synthetic cells. Following this strategy, orthogonal signaling cascades can be engineered by replacing NanoBiT with different enzymes that have split variants. For example, replacing NanoBiT with a split tobacco etch virus (TEV) protease allows for contact‐dependent chemical signaling in which a messenger molecule outside cells can enter cells only if it is processed by the reconstituted split TEV protease.^[^ ^65^ ^]^ Finally, the recent development of light‐activated SpyLigation^[^ ^66^ ^]^ which allows SpyTag–SpyCatcher bond formation only in the presence of light can make the activation of orthogonal signaling cascades dependent on each other, thus paving the way for designing signaling schemes with feedback and implementation of protein‐based circuits in contact interfaces between natural and synthetic cells.

## 4 Experimental Section

4.1

4.1.1

##### Cloning and Preparation of DNA Constructs

Plasmids containing the sequences of iLID and SspB Nano–mCherry were kindly gifted by Dr. Kristen Verhey (University of Michigan). Plasmids with DNA sequences of LgBiT and NLuc were generous gifts from Dr. Taekjip Ha (Harvard Medical School) and Dr. Gary Luker (University of Michigan), respectively. A maltose binding protein‐small ubiquitin‐like modifiers vector was a gift from Dr. Christopher Lima (Sloan Kettering Institute). Target sequences were amplified using Q5 high‐fidelity DNA polymerase (New England Biolabs). All primers are specified in Table S1, Supporting Information. Amplified DNA fragments were assembled using Gibson Assembly. See Section S1, Supporting Information, for more details about cloning and DNA preparation.

##### Protein Expression and Purification

All proteins were purified following the conventional affinity purification protocols. For more details, see Section S2, Supporting Information.

##### Luminescence Measurement

Luminescence measurements presented in Figure 2b and Figure S7, S8d, Supporting Information, were performed using a Synergy H1 (BioTek) multimode plate reader. All measurements were done using 1 s integration time and a gain of 130. For plots in Figure 2b, 8 μL mixture of different combinations of ST–SmBiT, SC–LgBiT, and SmBiT were prepared for a final concentration of 500 nM. The mixtures were incubated for 30 min at room temperature (RT) before being transferred to a 96‐well v‐shaped bottom plate. Next, 2 μL of 20‐fold diluted furimazine stock (Promega) in live cell substrate (LCS) dilution buffer (Nano–Glo assay, Promega) was added to each well. The plates were shaken for 10 s inside the plate reader and the measurement was performed right after adding the substrate to the solutions. Photon flux measurements presented in Figure S1, Supporting Information, were extracted from luminescence imaging data captured with an *in vivo* imaging system Lumina Series III (Perkin Elmer, Waltham, MA) and analyzed with Living Image 4.5.2. The integration time was set for 1 s with a binning factor of 2 and f‐stop value of 2. 50 μL solutions with different concentrations of NLuc and equimolar concentrations of ST–SmBiT and SC–LgBiT were transferred to a 96‐well clear flat bottom plate and incubated for 30 min in RT. Next, 12.5 μL 20‐fold diluted furimazine stock in LCS buffer was added to each well and the plates were shaken manually before the time series of luminescence images were captured every minute for 30 min.

##### SDS–PAGE and In‐Gel Imaging

For SDS–PAGE analyses presented in Figure 2d and Figure S2, Supporting Information, 10 μL solutions of 1 μM ST–SmBiT, SC–LgBiT, and a 1 μM equimolar mixture of ST–SmBiT and SC–LgBiT were prepared and incubated for 30 min at RT. The solutions were then mixed with 3.3 μL of 4x Laemmli buffer containing 10% 2‐mercaptoethanol and were incubated at 95 °C for 10 min. Then, the SDS–PAGE gel was run in a 4%–20% bis–tris polyacrylamide precast gel (Sigma Aldrich). The gel was stained using SimplyBlue stain (Invitrogen) and imaged by a Sapphire Biomolecular Imager (Azure biosystems) with 658/710 nm excitation/emission wavelengths.

##### GUV and SUV Preparation

In all experiments, GUVs were generated following the protocol described by Eaglesfield et al.^[^ ^67^ ^]^ with slight modifications^[^ ^68^ ^]^ detailed in Section S3, Supporting Information. Details of the preparation of SUVs and size exclusion chromatography purification of protein‐bound SUVs are presented in Section S4, Supporting Information.

##### Image Analysis

All GUV images were analyzed by ImageJ. The intensity profiles presented in Figure 3f and Figure S3, Supporting Information, were obtained by measuring the fluorescence intensity along the corresponding indicated lines in Figure 3d and Figure S3, Supporting Information, respectively. In addition, the background signal was measured and subtracted from the intensity values followed by signal intensity normalization to the maximum signal. For analyzing membrane recruitment of SspB–mCherry presented in Figure 3e, 4c,f, and Figure S5, S8d, Supporting Information, ImageJ Oval_profile plugin was used to measure the mCherry fluorescence intensity over 30 points equally spaced along the periphery of the GUV. Similarly, the fluorescence intensity of GUV lumen and background were measured and averaged.

For measuring SspB–mCherry recruitment to GUV–GUV interfaces presented in Figure 5c and Figure S9b, Supporting Information, the fluorescence intensity along five arbitrary lines crossing the GUV–GUV interfaces was measured and the average signal of three pixels centered around the peak intensity and the average signal of its 10 following points into the lumen of the receiver cell were calculated as the signal values associated with membrane and lumen, respectively. Since successful NanoBiT reconstitution can only occur between juxtaposed sender and receiver synthetic cells from random mixing of the two GUV populations, we chose only contact interfaces between sender and receiver cells (identified by FITC and mCherry, respectively) for our analysis. When there was no clear peak in the intensity profile (as in control cases), the average of three intensities centered around the point where the signal reached a plateau and the average signal of 10 following points into the lumen of a receiver cell were taken as membrane and lumen intensity values, respectively. All five measurements were then averaged and represented a single data point. For size distributions of GUVs analyzed in different experiments, please see Figure S10, Supporting Information.

##### Mathematical Modeling

A 1D mathematical model was developed to model the interaction of SspB–mCherry with membrane‐bound iLID inside a GUV under the effect of radial diffusion and chemical reaction between iLID and SspB. See Section 5 of SI for the details of the model.

##### Statistical Analysis

Experiments were performed in at least three independent replicates. The *p* values reported in Figure S1c, S6, S9b, Supporting Information, were calculated by Welch's *t* test and corrected by Bonferroni method in Figure S6, Supporting Information, with a factor of 3. The statistical significance between the ratio of membrane‐bound SspB–mCherry to luminal SspB–mCherry in the presence and absence of furimazine presented in Figure 4c,f was also calculated by Welch's *t* test. In addition, an R code was written and used to perform one‐way and two‐way analysis of variance (ANOVA) tests on data from analyzed images represented in Figure 3d, 5c and Figure S5b, S8d, Supporting Information, respectively, with Tukey's honest significance difference correction. Confidence intervals of regression lines presented in Figure 2c were calculated using OriginPro 2020b statistical analysis tool. All *p* values are listed in Table S2, Supporting Information. The data analysis code can be found here: GitHub—mhossein7/juxtacrine_signaling_code.

## Conflict of Interest

The authors declare no conflict of interest.

## Author Contributions

**Hossein Moghimianavval**: Conceptualization (lead); Data curation (lead); Formal analysis (lead); Writing—original draft (lead); Writing—review and editing (lead). **Kyle J. Loi**: Data curation (supporting). Sung‐Won Hwang: Writing—review and editing (supporting). **Yashar Bashirzadeh**: Writing—review and editing (supporting). **Allen P. Liu**: Conceptualization (lead); Funding acquisition (lead); Project administration (lead); Resources (lead); Supervision (lead); Writing—original draft (lead); Writing—review and editing (lead).

## Supporting information

Supplementary Material

## Data Availability

The data that support the findings of this study are available from the corresponding author upon reasonable request.
