# Supplementary material for: Light‐Based Juxtacrine Signaling Between Synthetic Cells
Source: Small Sci. 2024 Oct 30;5(1):2400401. doi: 10.1002/smsc.202400401 (PMC11935020; doi:10.1002/smsc.202400401)
Supplement: Supplementary file 1 — Supplementary Material [file SMSC-5-2400401-s001.pdf]

Supporting Information

Light-based juxtacrine signaling between synthetic cells

Hossein Moghimianavval[1], Kyle J. Loi[2,3], Sung-Won Hwang[4], Yashar Bashirzadeh[1], Allen P. Liu[1,3,5,6] *

[1] Department of Mechanical Engineering, University of Michigan, Ann Arbor, MI, USA

[2] Neuroscience Program, University of Michigan, Ann Arbor, MI, USA

[3] Cellular and Molecular Biology Program, University of Michigan, Ann Arbor, MI, USA

[4] Department of Chemical Engineering, University of Michigan, Ann Arbor, MI, USA

[5] Department of Biomedical Engineering, University of Michigan, Ann Arbor, MI, USA

[6] Department of Biophysics, University of Michigan, Ann Arbor, MI, USA

* Corresponding author: Allen P. Liu, allenliu@umich.edu

1. Cloning and preparation of DNA constructs

First, the sequences encoding for LgBiT and SpyCatcher003 were amplified from the original LgBiT plasmid and sCatch-GFP plasmid (Addgene #18690241,58), respectively, and were cloned into a pET28b vector to create SpyCatcher-LgBit-6xHis (SC-LgBiT) construct. To generate the SpyTag-SmBiT construct, first, the MBP sequence from MBP-SUMO vector with an N-terminal 6xHis tag was amplified. A g-Block encoding for SmBiT-SpyTag with flexible linkers between SmBiT and SpyTag and at the N-terminus of the SmBiT was ordered from Integrated DNA Technologies (IDT). Next, the g-Block and the amplified MBP were cloned into the pET28b vector to generate 6xHis-MBP-SmBiT-SpyTag (ST-SmBiT) construct. The control construct SmBiT lacking the SpyTag domain was generated by a two-step PCR where a C-terminal SmBiT domain was added to the MBP sequence before cloning into the pET28b vector. Similarly, the iLID construct was generated by cloning amplified MBP-SUMO and iLID sequences and cloning them into pET28b vector to create MBP-SUMO-iLID-6xHis. The amplified SspB-mCherry fragment was cloned into a pGEX-6P-1 vector (from our previous studies[1,2]) to generate GST-mCherry-SspB. Lastly, the NLuc and MBP sequences were amplified and cloned into pET28b vector to generate NLuc-MBP-6xHis.

All cloning sequences were verified by Sanger sequencing (Eurofins). The assembled DNA constructs were purified from XL10-Gold ultracompetent cells (Agilent) using miniprep kits (Qiagen).

2. Protein expression and purification

SC-LgBiT and iLID were purified following the conventional His-purification protocol reported elsewhere[3,4]. Plasmids encoding for the SC-LgBiT or iLID were transformed into BL21-DE3-pLysS competent cells. Single colonies were picked and grown in 5 mL LB broth supplemented with 50 µg/mL kanamycin overnight at 37 °C shaking at 220 rpm. Next, the culture was diluted in 1 L LB supplied with 0.8% w/v glucose and 50 µg/mL kanamycin and was grown at 37 °C shaking at 220 rpm until A600 reached 0.5-0.6. The culture was then induced with 0.42 mM isopropyl β-D-1-thiogalactopyranoside (IPTG) and incubated at 30 °C with constant shaking at 200 rpm for 4-5 h. The cells were next harvested by centrifugation at 5000 g for 10 min at 4 °C and then resuspended in 30 mL lysis buffer containing 50 mM Tris-HCl (pH 7.4), 300 mM NaCl, 30 mM Imidazole, and 1 mM 4-(2-aminoethyl) benzenesulfonyl fluoride hydrochloride (AEBSF). The cells were then lysed by a sonicator (Branson Sonifier 450) and the lysate was centrifuged

at 30000 g for 25 min at 4 °C. The supernatant was then run through an equilibrated 1 mL HisTrap column (Cytiva) on an AKTA start fast protein liquid chromatography (FPLC) system. Next, the column was washed with 15 column volume washing buffer containing 50 mM Tris-HCl (pH 7.4), 300 mM NaCl, and 50 mM Imidazole. The protein was then eluted by 10 column volumes of elution buffer composed of 50 mM Tris-HCl (pH 7.4), 300 mM NaCl, and 300 mM Imidazole and was collected in 1 mL fractions. The quality of protein purification in each fraction was assessed by SDS-PAGE analysis and the fractions with high concentrations of the protein were pooled and dialyzed against 1 L PBS overnight at 4 °C. The protein concentration was measured using NanoDrop (Thermo Fisher Scientific) and the protein was aliquoted and stored at -80 °C until use. An individual batch of iLID was labelled with FITC using a FITC Conjugation Kit (Invitrogen) following the manufacturer protocol and was immediately used for the experiment presented in **Fig. S3**.

NLuc, ST-SmBiT, and SmBiT were purified following the same steps described above with the following changes. After harvesting, the cells were resuspended in 30 mL lysis buffer containing 20 mM Tris-HCl (pH 7.4), 300 mM NaCl, 1 mM EDTA, and 1 mM AEBSF and then were lysed by sonication. The lysate was centrifuged at 30000 g for 25 min at 4 °C, and the supernatant was passed through an equilibrated 1 mL MBPTrap (Cytiva) on an AKTA start FPLC system. The bound protein was washed with 15 column volumes of washing buffer containing 20 mM Tris-HCl (pH 7.4) and 300 mM NaCl. The protein was then eluted with 10 column volumes of elution buffer composed of 20 mM Tris-HCl (pH 7.4), 300 mM NaCl, and 10 mM maltose. The purification quality was assessed by SDS-PAGE and the fractions with high concentrations of the protein were pooled and dialyzed against 1 L PBS overnight at 4 °C. The protein concentration was next measured using NanoDrop and the protein was aliquoted and stored at -80 ℃ until use.

Lastly, SspB-mCherry was purified by following conventional GST-purification protocols described elsewhere[1,2]. A single colony of transformed BL21-DE3-pLysS competent cells was picked and grown in 5 mL LB supplemented with 100 µg/mL ampicillin overnight at 37 °C shaking at 220 rpm. Next, the culture was diluted in 1 L LB supplied with 100 µg/mL ampicillin and the cells were grown at 37 □C shaking at 220 rpm until A600 reached 0.5-0.6. The culture was then induced with 0.1 mM IPTG and incubated at 30 °C with constant shaking at 200 rpm for 4-5 h. The cells were next pelleted by centrifugation at 5000 g for 10 min at 4 °C and then resuspended in 30 mL lysis buffer composed of PBS and 1% Triton-X100, 1 mM AEBSF, and 1 mM DTT. The cells were lysed by sonication, and the cell lysate was centrifuged at 30,000 g for

25 min at 4 °C and the supernatant was loaded onto an equilibrated 1 mL GSTrap (Cytiva) on an AKTA start FPLC system. The column was then washed with 15 column volumes of washing buffer containing PBS and 1 mM DTT, and the bound protein was eluted by 10 column volumes of elution buffer composed of 50 mM Tris-HCl (pH 7.4), 20 mM glutathione, and 1 mM DTT. The purification quality was analyzed with SDS-PAGE and fractions with high concentration of protein were pooled and dialyzed against 1 L PBS overnight at 4 °C. The protein concentration was measured using NanoDrop and the protein was aliquoted and stored at -80 °C until use.

3. GUV preparation

Appropriate amounts of lipids 1-palmitoyl-2-oleoyl-glycero-3-phosphocholine (POPC) and 1,2-dioleoyl-sn-glycero-3-[(N-(5-amino-1-carboxypentyl) iminodiacetic acid) succinyl] (nickel salt) (DGS-NTA (Ni)) (Avanti Polar Lipids) in chloroform were transferred to a glass vial for a final concentration of 500 µM lipid in oil with 95% POPC and 5% DGS-NTA (Ni) composition. The chloroform was then evaporated under a gentle stream of argon. Next, an appropriate amount of light mineral oil (Sigma-Aldrich, Cat#: M5904) was added to the lipid film and vortexed thoroughly to ensure the lipid is dissolved. The lipid-in-oil solution was incubated at 50 °C for 20 min before being vortexed again. Next, 300 µL of the lipid-in-oil solution was gently layered on top of 400 µL outer solution composed of 50 mM Tris-HCl (pH 7.4) and 300 mM glucose in a 1.5 mL microcentrifuge tube, and the oil-water interface was incubated for 1 h at RT. In a separate 1.5 mL microcentrifuge tube, the inner solution (details in the following subsections) was mixed with 600 µL lipid-in-oil solution and the solution was pipetted up and down for 1 min to make a uniform emulsion. The emulsion was then added gently on top of the oil layer, and the tube containing the emulsion and the oil-water interface was centrifuged at 2500 g for 10 min at RT. Next, the top 900 µL of lipid-in-oil solution was removed by aspiration followed by the aspiration of the outer solution until the remaining outer solution volume was around 100 µL. The GUVs were then resuspended in the remaining outer solution by gently pipetting up and down.

3.1. iLID-SspB dimerization by external illumination

GUVs were made by encapsulating 20 µL inner solution containing 50 mM Tris-HCl (pH 7.4), 300 mM glucose, 450 nM iLID-6xHis, 100 nM SspB-mCherry, and 10% v/v OptiPrep (Sigma-Aldrich). After collection, 50 µL of GUV solution was transferred to a 96-well clear flat bottom

plate and kept in the dark for 30 min before illumination and epifluorescence imaging using a Nikon TiE inverted microscope equipped with an oil immersion Plane Apochromat 60× objective (NA 1.40), a sCMOS camera (Flash 4.0; Hamamatsu Photonics, Japan), and an HBO 100 W/2 mercury bulb. Next, GUVs were illuminated with an excitation wavelength of 488 nm and intensity of *ca.* 70 mW/cm^2 for 15 min before single images of SspB-mCherry were taken at an excitation wavelength of 561 nm using ImageJ image acquisition software (NIH).

3.2. iLID-SspB dimerization by NLuc

For experiments with NLuc inside synthetic cells, GUVs were generated by encapsulating 20 µL inner solution containing 50 mM Tris-HCl (pH 7.4), 300 mM glucose, 450 nM iLID-6xHis, 100 nM SspB-mCherry, 500 nM NLuc, and 10% v/v OptiPrep. After GUVs were collected, 80 µL of GUV solution was transferred to a 96-well clear flat bottom plate and incubated in the dark for 30 min. Next, 20 µL LCS buffer containing a 20-fold dilution of furimazine stock was added to the well and epifluorescence images of SspB-mCherry were taken immediately. A 100-fold dilution of furimazine stock was added to the well every 15 min for a total of three times during imaging.

For experiments with NLuc attached to the outer membrane of synthetic cells, GUVs were made by encapsulating 20 µL inner solution containing 50 mM Tris-HCl (pH 7.4), 300 mM glucose, 450 nM iLID-6xHis, 100 nM SspB-mCherry, and 10% v/v OptiPrep. After GUVs were collected, 500 nM NLuc was added to the GUV solution and GUVs were incubated at RT for 30 min. Next, 900 µL outer solution was added to the GUV solution and the solution was centrifuged at 2500 g for 10 min at RT. Next, 900 µL of outer solution was removed by gentle pipetting. The GUV pellet was then resuspended by gently pipetting up and down and 80 µL of GUV solution was transferred to a 96 well clear flat bottom plate. Then, 20 µL LCS buffer containing a 20-fold dilution of furimazine stock was added to the well and epifluorescence images of SspB-mCherry were taken immediately. A 100-fold dilution of furimazine stock was added to the well every 15 min for a total of three times during imaging.

3.3 iLID-SspB dimerization by NanoBiT

Sender cells were generated by encapsulating 20 µL inner solution containing 50 mM Tris-HCl (pH 7.4), 500 mM KCl, 12.5 µM Fluorescein isothiocyanate (FITC)-dextran, and 10% v/v OptiPrep in an outer solution containing 50 mM Tris-HCl (pH 7.4) and 1 M glucose. Similarly,

receiver cells were made by encapsulating 20 µL inner solution containing 50 mM Tris-HCl (pH 7.4), 500 mM KCl, 450 nM iLID-6xHis, 100 nM SspB-mCherry, and 10% v/v OptiPrep in an outer solution containing 50 mM Tris-HCl (pH 7.4) and 1 M glucose. After collecting GUVs, 5 µM SC-LgBiT and ST-SmBiT were added to sender and receiver cells, respectively, in individual 1.5 mL microcentrifuge tubes and GUV solutions were incubated at RT for 30 min. Next, the GUVs were washed by adding 900 µL outer solution to each tube followed by centrifugation at 2500 g for 10 min at RT. Then, 900 µL of supernatant from each tube was removed gently by pipetting. Each population of GUVs was then resuspended and a 100 µL 1:1 mixture of sender cells and receiver cells was made and transferred to a 96-well clear flat bottom plate. Next, 100 µL 1 M NaCl was added to the well to induce hyper-osmotic condition and the GUVs were incubated for 30 min in the dark to let membrane-membrane interfaces and NanoBiT form. GUV images were taken using an oil immersion Plan-Apochromat 60 x/1.4 NA (Olympus) objective on an inverted microscope (Olympus IX-81) equipped with an iXON3 EMCCD camera (Andor Technology), National Instrument DAQ-MX controlled laser (Solamere Technology), and a Yokogawa CSU-X1 spinning disk confocal unit. Images were acquired using MetaMorph (Molecular Devices). Single images of FITC and SspB-mCherry were taken at excitation wavelengths of 488 and 561 nm, respectively. Then, 50 µL LCS buffer containing a 20-fold dilution of furimazine stock was added to the well, and images of FITC and SspB-mCherry were taken immediately. A 100-fold dilution of furimazine stock was added to the well every 15 min during imaging.

4. SUV preparation and size exclusion chromatography

100 nm-sized SUVs were prepared by the rehydration method followed by extrusion detailed elsewhere[3,4]. A solution of 5 mM vesicles of 90% DOPC and 10% DGS-NTA (Ni) were made by mixing appropriate amounts of DOPC and DGS-NTA (Ni) lipid stocks dissolved in chloroform in a 20 mL glass vial with a screw cap. The mixture was then dried using a gentle stream of argon to form a lipid film. The dried lipid was then incubated at a desiccator for 1 h to ensure removal of residual organic solvent. Next, 500 µL PBS was added to the lipid film and the lipid was rehydrated by vortexing at maximum speed for 1 min. The dissolved lipid was then passed through a 100 nm polycarbonate membrane (Hamilton) for 11 times using an Avanti mini-extruder.

The size exclusion chromatography (SEC) column was prepared by packing a liquid chromatography column with Sepharose 4B (Sigma-Aldrich). First, 50 µL of 5 mM SUVs labeled

with 1,2-dioleoyl-sn-glycero-3-phosphoethanolamine-N-(7-nitro-2-1,3-benzoxadiazol-4-yl) (ammonium salt) (NBD-PE) (Avanti Polar Lipids) was loaded onto the column and 100 µL elution fractions were collected to determine the required volume for eluting SUVs. Next, 50 µL of 5 mM unlabeled SUVs were mixed with 4 µM SC-LgBiT and incubated for 30 min at RT. The mixture was then loaded onto the SEC column and 100 µL fractions eluted with PBS were collected. The fractions containing the SUVs were kept for further experiments. Protein-bound SUVs were prepared immediately before the experiment.

5. Mathematical modeling

The model considers the interaction of SspB-mCherry with membrane-bound iLID inside a GUV with radius R under the effect of radial diffusion and chemical reaction between iLID and SspB. For simplicity, the angular diffusion was neglected. The diffusion along the radius of the GUV was mathematically modeled with the following equation:

$$\frac{\partial C}{\partial t} = D\frac{\partial^2 C}{\partial r^2}$$

Where *C*, *r*, *D*, and *t* represent the concentration of SspB-mCherry, the radius, the diffusion coefficient, and time, respectively. The equation requires an initial condition and two boundary conditions. A Neumann boundary condition at the *r* = 0 was assumed:

$$\frac{\partial C}{\partial t}|_{r=0} = 0$$

This boundary condition implies that the flow of diffusion will always be from the center of the GUV towards the membrane. At the membrane (*r* = R), the boundary condition is coupled to the chemical reaction between SspB and iLID. If the concentration of iLID and SspB-iLID dimer is represented by *L* and *M*, respectively, the chemical reaction can be modeled with the following set of equations:

$$\frac{\partial C}{\partial t}|_{r=R} = k_{-}M - k_{+}C|_{r=R}L$$

$$\frac{dL}{dt} = k_{-}M - k_{+}C|_{r=R}L$$

$$\frac{dM}{dt} = k_{+}C|_{r=R}L - k_{-}M$$

in which, k_+ and k_- represent the association and dissociation rate constants of iLID and SspB dimerization. For simplicity, it was assumed that under light illumination, only k_+ changes. However, the ratio of k_+ to k_- follows experimental data reported by Guntas *et al*.[5] That is, k_+ in light is 36 times higher than k_+ in the dark. Since the above partial differential equation (PDE) has a boundary condition in the form of a set of ordinary differential equations (ODE), it cannot be solved analytically. Therefore, a finite difference model was developed to solve the PDE and ODEs simultaneously using Matlab. With small timesteps, it was reasoned that in each step, the ODE can be solved first, thus generating a fixed boundary condition for PDE in the next step, and this cycle can be repeated until the solution is convergent.

5.1. Finite-difference modeling of ODEs

Assuming u_0, u_1, and u_2 represent the concentration of SspB, iLID, and SspB-iLID dimer at the membrane, respectively, at time t, after a small timestep Δt, the new concentrations can be calculated by the following equations:

$$\frac{u_0^1 - u_0}{\Delta t} = k_- u_2 - k_+ u_0 u_1$$

$$\frac{u_1^1 - u_1}{\Delta t} = k_- u_2 - k_+ u_0 u_1$$

$$\frac{u_2^1 - u_2}{\Delta t} = k_+ u_0 u_1 - k_- u_2$$

in which, the variables with superscript 1 represent the value of the variable at time t + Δt.

5.2. Finite-difference modeling of PDEs

The diffusion PDE can be reduced to algebraic equations using a finite-difference approach assuming that the GUV radius is divided into N equal elements. If C_n represents the concentration of SspB in the nth element, the PDE will be reduced to the following equation:

$$\frac{C_n^{t+1} - C_n^t}{\Delta t} = D \frac{C_{n+1}^t - 2C_n^t + C_{n-1}^t}{h^2}$$

$$C_n^{t+1} - C_n^t = F(C_{n+1}^t - 2C_n^t + C_{n-1}^t)$$

where

$$F = \frac{D\Delta t}{h^2}$$

$$h = \frac{R}{N}$$

and t+1 and t superscripts stand for the concentration of SspB at the time t + Δt and t, respectively. At the first element, we can apply the Neumann boundary condition which gives us the following:

$$C_1^{t+1} = C_1^t + 2F(C_2^t - C_1^t)$$

To apply the Neumann boundary condition at r = R, the rate of change in concentration of SspB at this element is considered to be constant and can be calculated from the set of ODEs at r = R:

$$\frac{\partial C}{\partial t}|_{r=R} = k_- M^t - k_+ C_N^t L^t = B$$

where C_N stands for the concentration of SspB at the Nth element. Applying this boundary condition to the finite-difference model results in the following:

$$C_N^{t+1} = C_N^t + 2F(C_{N-1}^t - C_N^t + Bh)$$

If all C_n at time t and t + Δt are represented in column vectors $\mathbf{C^t}$ and $\mathbf{C^{t+1}}$, respectively, then the $\mathbf{C^{t+1}}$ can be found from the relationship between $\mathbf{C^t}$ and $\mathbf{C^{t+1}}$:

$$\boldsymbol{C^{t+1}} = \boldsymbol{AC^t}$$

in which **A** is the following N by N matrix:

$$\boldsymbol{A} = \begin{bmatrix} 1-2F & 2F & 0 & \dots & \dots & \dots & 0 \\ F & 1-2F & F & 0 & \dots & \dots & 0 \\ 0 & F & 1-2F & F & 0 & \dots & 0 \\ \vdots & \ddots & \ddots & \ddots & \ddots & \ddots & \vdots \\ 0 & \dots & \dots & F & 1-2F & F & 0 \\ 0 & \dots & \dots & 0 & F & 1-2F & F \\ 0 & \dots & \dots & 0 & 0 & 2F & 1-2F \end{bmatrix}_{N\times N}$$

In each step, after finding the value of $\mathbf{C^{t+1}}$, 2FBh is added to the C_N^{t+1} to account for the boundary condition. This approach was implemented in a Matlab code to solve the diffusion PDE coupled with boundary condition ODEs with the initial condition of 0.05, 1.5, and 0 for the concentration of SspB in each element, iLID at the Nth element, and SspB-iLID dimer at N^{th} element, respectively. The results presented in **Fig. 3b** were plotted using Matlab. The bar

graph in **Fig. 3c** was made by obtaining the final values of the solution for luminal SspB and SspB-iLID dimer. Simulation code can be found here: GitHub - mhossein7/juxtacrine_signaling_code

6. Mathematical analysis of iLID and NTA (Ni) saturation

First, we note that the ratio of membrane area to the GUV volume scales with inverse of the GUV radius:

$$A = 4\pi r^2, V = \frac{4}{3}\pi r^3$$

$$\frac{A}{V} = \frac{3}{r}$$

Given the ratio of encapsulated iLID to SspB which is 4.5, there will always be 4.5 times more iLID molecules present in the GUV compared to SspB (no iLID saturation). However, because volume grows faster than the area when the radius increases, at some point, the area of the GUV will not be sufficient to contain all iLID molecules (NTA (Ni) saturation), thus leaving iLID in the lumen to dimerize with SspB (but unlikely since light is only emitted at the membrane-membrane interface). This could jeopardize the analysis as iLID saturation will cause lower membrane to lumen fluorescence ratio. We find this threshold and demonstrate that given our GUV sizes, we do not have this problem:

$$N_{NTA} = 0.05\frac{4\pi r^2}{0.6 \times 10^{-18}}$$

$$N_{iLID} = 450 \times 10^{-6}\frac{4\pi r^3}{3} \times N_A$$

$$\frac{N_{NTA}}{N_{iLID}} = \frac{5 \times 10^{16}}{9r \times 10^{18}} = \frac{5}{900\, r}$$

where r is the GUV radius and is in meters, N_{NTA} and N_{iLID} represent the number of NTA (Ni) lipids to which iLID can bind (note that we consider that the GUV membrane contains 5% NTA (Ni)) and the number of iLID molecules, respectively. Additionally, N_A is the Avogadro's number and $0.6\, nm^2$ is considered the average area of a single lipid molecule. This analysis indicates that the threshold where iLID saturates at the membrane occurs for GUVs with radii higher than $\sim 5\, mm$. Therefore, we conclude that for our GUVs which have sizes well below this limit (**Fig. S10**), the effect of size variation on the ratio of membrane to lumen signal is negligible.

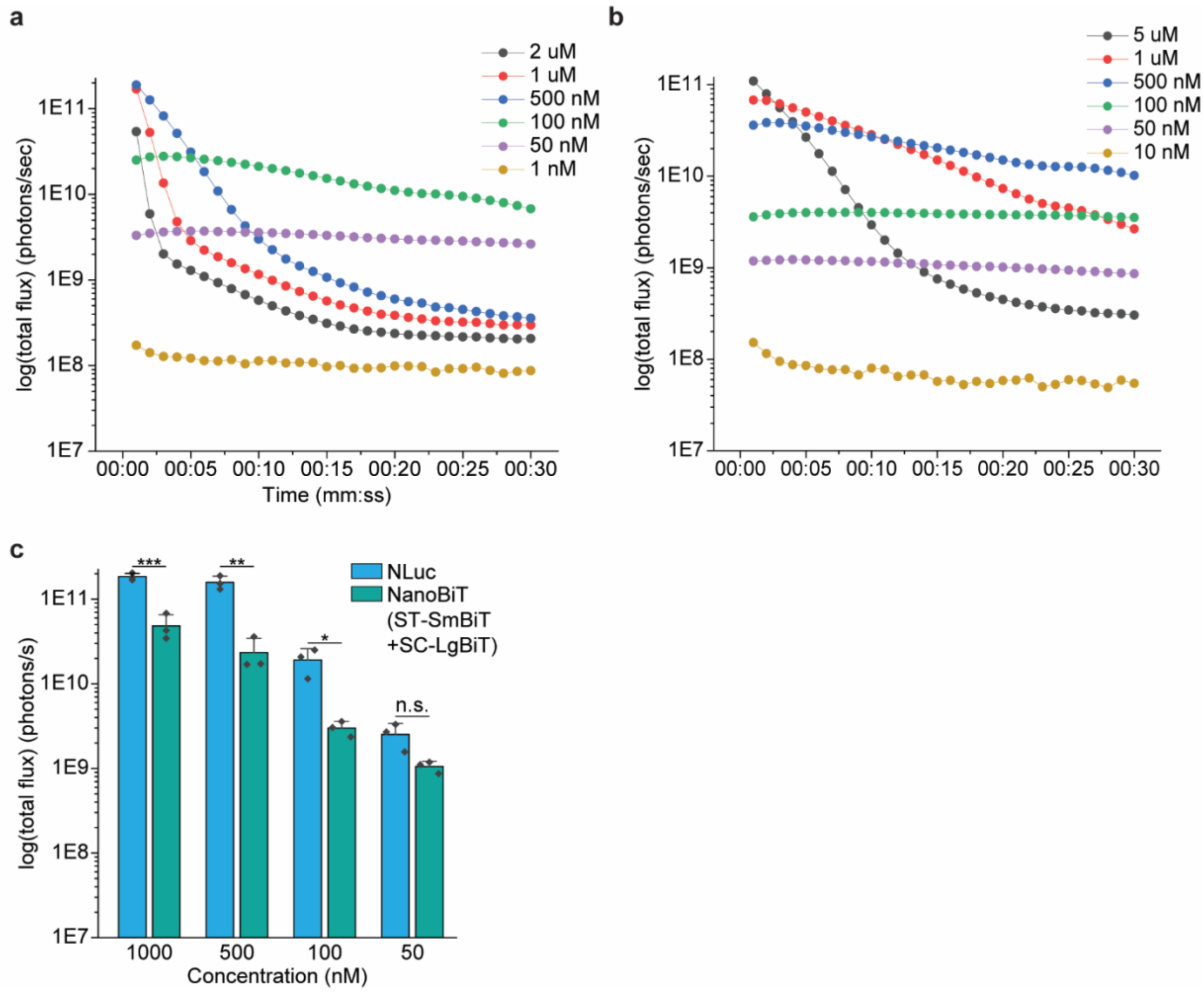

Figure S1| Luminescence imaging of NLuc and reconstituted NanoBiT reactions. Representative time series depicting quantification of luminescence imaging of 50 μL reactions with different NLuc **(a)** or NanoBiT **(b)** concentrations in the presence of 1:4 dilution of live cell substrate (LCS) buffer containing 20-fold dilution of furimazine stock. **c,** Bar plots comparing the maximum luminescence at different concentrations of NLuc and NanoBiT. Error bars show the standard deviation. *p*-values are calculated using one-tailed Welch's t-test, $n = 3$. ***, **, *, and n.s. represent $p < 0.001$, $p < 0.01$, $p < 0.05$, and not significant, respectively.

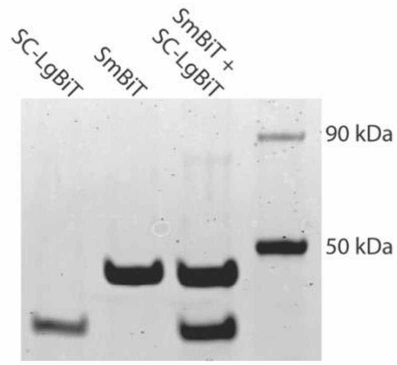

Figure S2| Absence of SpyTag-SpyCatcher interaction results in no dimer NanoBiT formation. In-gel fluorescence imaging of the Coomassie-stained SC-LgBiT (lane 1), SmBiT (lane 2), the mixture of SC-LgBiT and SmBiT (lane 3), and the ladder (lane 4).

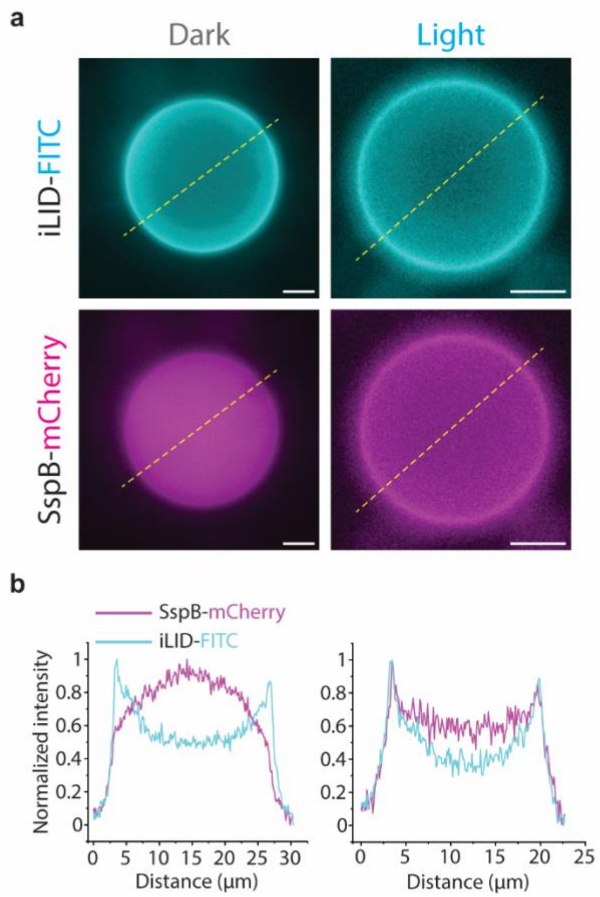

Figure S3| iLID-FITC is membrane-bound in synthetic cells made of POPC and DGS-NTA (Ni) regardless of light stimulation. a, Representative fluorescence images of synthetic cells encapsulating iLID-FITC (cyan) and SspB-mCherry (magenta) in the dark (left) or exposed to 488 nm light for 15 min (right). Scale bars: 5 µm. **b,** Intensity profiles of SspB-mCherry (magenta) and iLID-FITC (cyan) along the dashed yellow lines indicated in **a** reveal iLID-FITC localization to the membrane and SspB-mCherry membrane translocation due to the light exposure. Each plot corresponds to the image above in **a**.

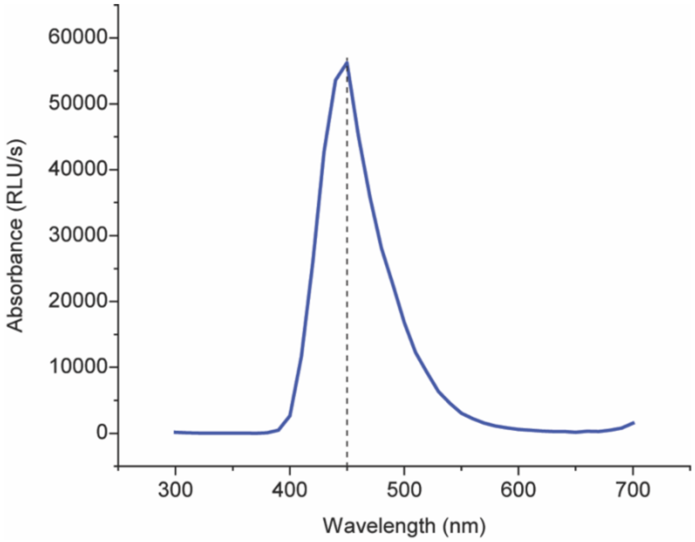

Figure S4| NLuc luminescence peaks at 450 nm. Luminescence emission spectrum of 500 nM NLuc in the presence of 1:4 dilution of LCS buffer containing 20-fold dilution of furimazine stock.

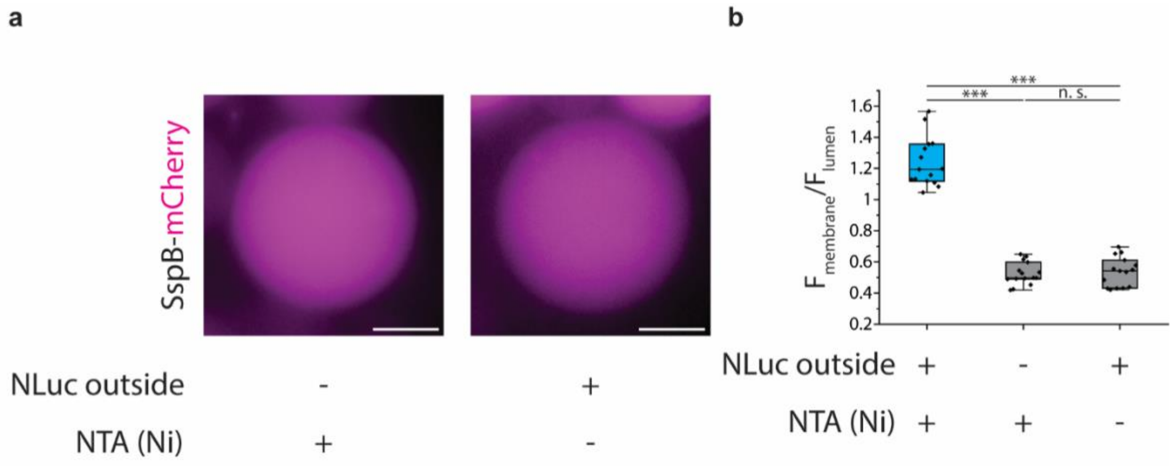

Figure S5| Absence of NLuc or NTA (Ni) results in no SspB-mCherry membrane recruitment. a, Representative fluorescence image of a GUV encapsulating SspB-mCherry without addition of NLuc to its outer solution (left) or without including DGS-NTA (Ni) lipid in its membrane composition (right). Scale bars: 10 µm **b,** Box plot comparing the ratio of SspB-mCherry signal at the GUV membrane to the luminal SspB-mCherry signal in POPC and DGS-NTA (Ni) GUVs with or without outer membrane-bound NLuc or POPC GUVs with NLuc. The data shows the average ratio of SspB-mCherry signal at the membrane of the GUV to the luminal SspB-mCherry signal with background subtraction for 30 points for ten different GUVs. The box represents the 25–75th percentiles and the median is indicated. The whiskers show the minimum and maximum data points, n = 15 from 4 independent experiments. p-values are calculated using two-way ANOVA test and corrected using Tukey's Honest Significant Difference. n.s. denotes not significant and *** represents $p < 0.001$.

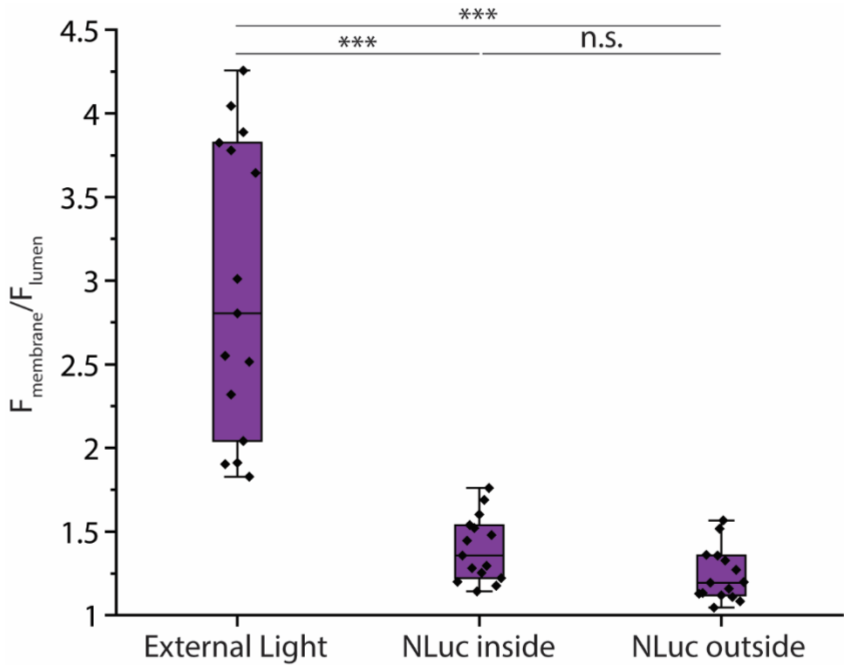

Figure S6| SspB-mCherry membrane recruitment through iLID activation via an external light source is significantly stronger than NLuc-mediated iLID activation. Box plot comparing the ratio of SspB-mCherry fluorescence signal at GUV membrane to the luminal SspB-mCherry signal for GUVs encapsulating 450 nM iLID and 100 nM SspB-mCherry excited by an external light source (see Methods) for 15 minutes, 500 nM encapsulated NLuc, or 500 nM NLuc attached to their outer membrane. The data shows the average ratio of SspB-mCherry signal at the membrane of the GUV to the luminal SspB-mCherry signal with background subtraction for 30 points for ten different GUVs. The box represents the 25–75th percentiles, and the median is indicated. The whiskers show the minimum and maximum data points, $n = 15$ from 3 independent experiments. p-values are calculated using one-way ANOVA test and corrected using Tukey's Honest Significant Difference. n.s. denotes not significant and *** represents $p < 0.001$.

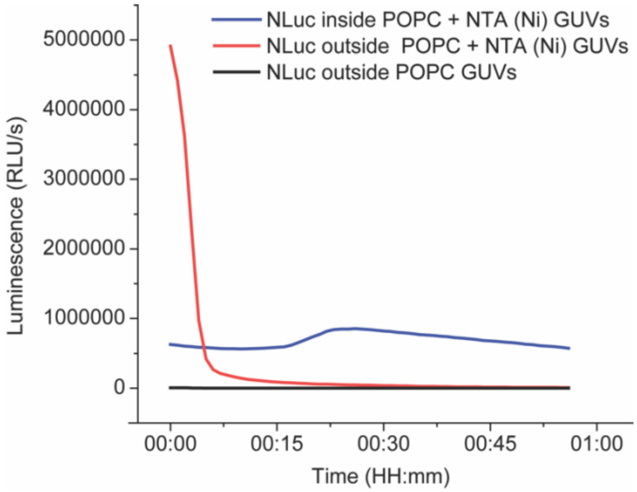

Figure S7| Encapsulated membrane-bound NLuc demonstrates a different reaction kinetics compared to NLuc attached to the outer membrane of synthetic cell. Representative luminescence readout from 100 µL solutions of GUVs with NLuc either attached to their inner or outer membranes or POPC GUVs washed after incubation with NLuc. Each solution was mixed with 25 µL of LCS buffer containing 20-fold dilution of furimazine stock right before luminescence measurement.

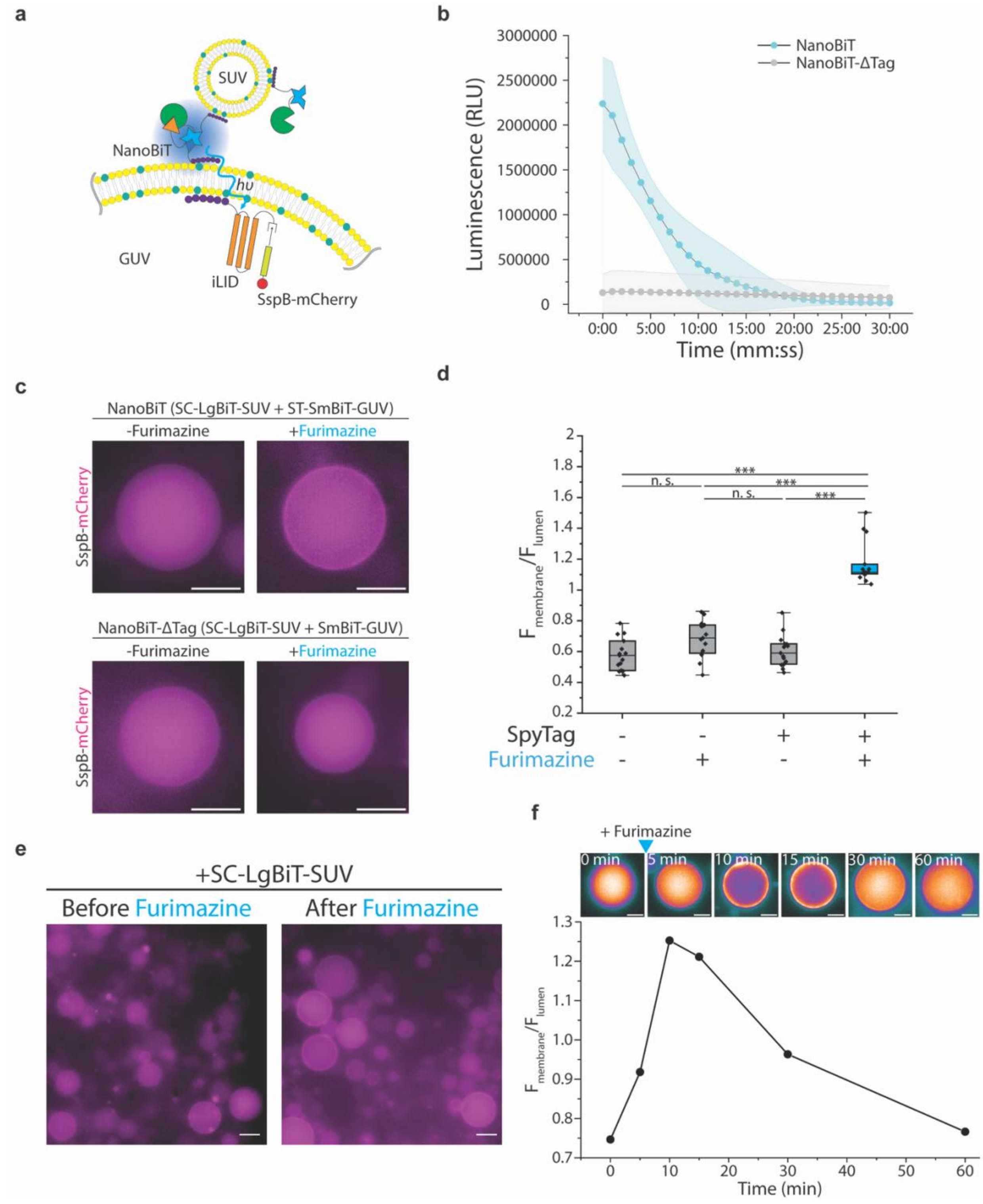

Figure S8| Reconstitution of NanoBiT by SUVs bound to SC-LgBiT as extracellular signals. a, Schematic illustrating membrane-bound iLID activation by reconstituted NanoBiT between a GUV harboring ST-SmBiT and an SUV harboring SC-LgBiT. **b,** Luminescence readout from protein pairs on GUV-SUV that reconstitute NanoBiT or NanoBiT without SpyTag

on SmBiT (i.e., no functional NanoBiT reconstitution). **c,** Representative images of GUV-SUV that reconstitute NanoBiT or NanoBiT without SpyTag in the absence or presence of furimazine. Scale bar: 10 µm. **d,** Boxplots comparing the ratio of SspB-mCherry fluorescence intensity at the GUV membrane to the luminal fluorescence intensity in GUVs harboring ST-SmBIT and encapsulating SspB-mCherry with added SUVs with ST-SmBiT or SmBiT with or without furimazine**.** The box represents the 25–75th percentiles, and the median is indicated. The whiskers show the minimum and maximum data points, $n = 15$ from 3 independent experiments. p-values are calculated using two-way ANOVA and corrected using Tukey's Honest Significant Difference. *** represents $p < 0.001$. **e,** Representative images of a cohort of receiver GUVs incubated with SC-LgBiT-SUVs (*i.e.*, sender cells) for 30 min. Images were taken before and 15 minutes after the addition of furimazine. Scale bar: 10 µm. **f,** Representative fluorescence images of SspB-mCherry in a receiver GUV mixed with SC-LgBiT-SUVs taken over 60 mins after the addition of furimazine. SspB-mCherry is shown using a heatmap lookup table scale for a better illustration of membrane-bound signals. The ratio of SspB-mCherry signal at the membrane to the luminal signal is also shown for each time point. Scale bar: 5 µm.

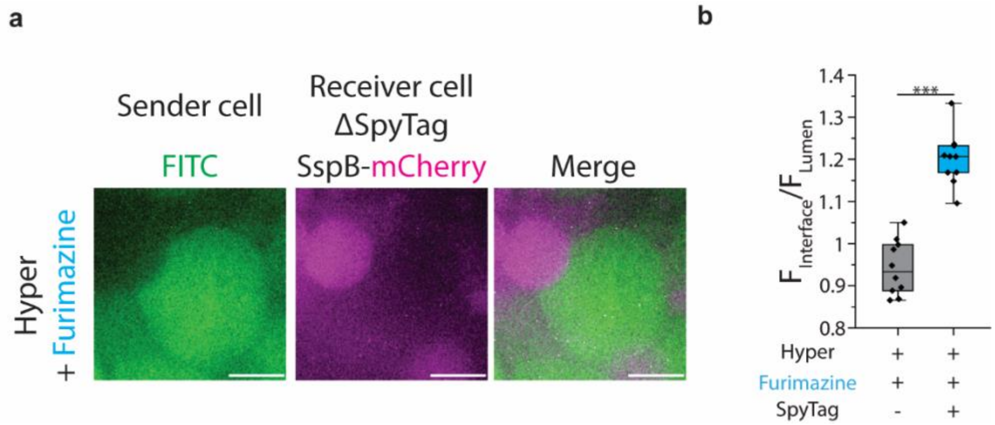

Figure S9| Deletion of SpyTag domain from ST-SmBiT on receiver cells results in no SspB-mCherry translocation to the membrane of receiver cells. a, Representative confocal images of FITC (green) encapsulated in a sender cell and SspB-mCherry (magenta) encapsulated in a receiver cell. Scale bars: 10 μm. **b,** Box plots comparing the ratio of the SspB-mCherry fluorescence intensity at the membrane of a receiver cell to the luminal SspB-mCherry fluorescence intensity in the presence or absence of SpyTag domain in ST-SmBiT. The data shows the average ratio of SspB-mCherry signal to the luminal SspB-mCherry signal for membrane-membrane interfaces of sender and receiver cells for at least three different receiver cells. The box represents the 25–75th percentiles, and the median is indicated. The whiskers show the minimum and maximum data points, $n = 10$ from 6 independent experiments. p-value is calculated using two-tailed Welch's test. *** represents $p < 0.001$.

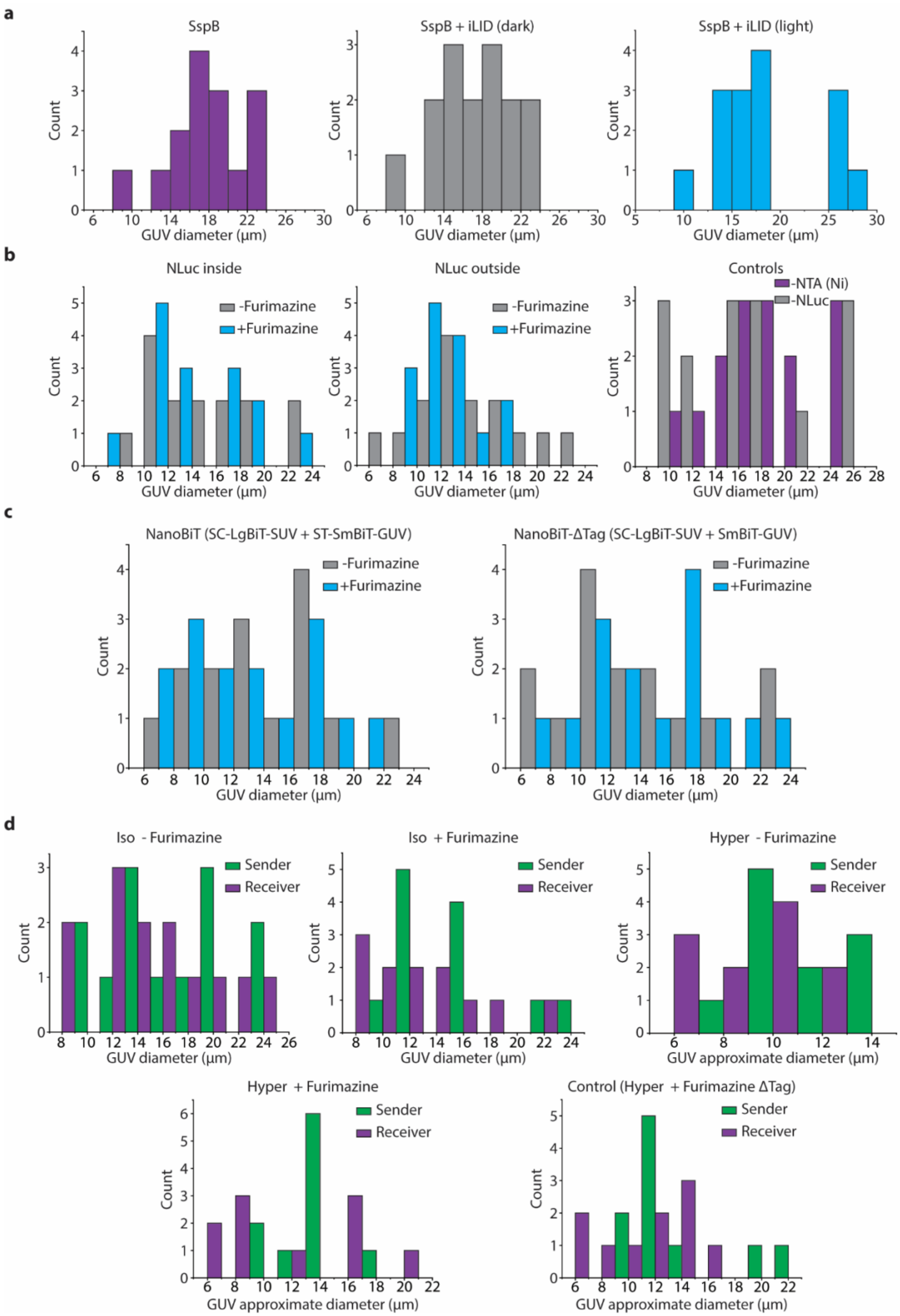

a
SspB
SspB + iLID (dark)
SspB + iLID (light)
Count
GUV diameter (µm)
b
NLuc inside
NLuc outside
Controls
-Furimazine
+Furimazine
-NTA (Ni)
-NLuc
Count
GUV diameter (µm)
c
NanoBiT (SC-LgBiT-SUV + ST-SmBiT-GUV)
NanoBiT-ΔTag (SC-LgBiT-SUV + SmBiT-GUV)
-Furimazine
+Furimazine
Count
GUV diameter (µm)
d
Iso - Furimazine
Iso + Furimazine
Hyper - Furimazine
Hyper + Furimazine
Control (Hyper + Furimazine ΔTag)
Sender
Receiver
Count
GUV diameter (µm)
GUV approximate diameter (µm)

Figure S10| GUV size distribution for different experiments. a, GUV size distribution for data presented in Fig. 3e. **b**, GUV size distribution for data presented in Figs. 4c, 4f, and S5b. **c**, GUV size distribution for data presented in Fig. S8d. **d**, GUV size distribution for data presented in Figs. 5c and S9b.

Table S1: List of all generated constructs and primers used for cloning

Construct	Fragment	Primers	Template DNA (Source)
pET28b-His-MBP-SmBiT-SpyTag	Vector	**Following primers were used for amplifying vector for all pET28b constructs**<br>**FWD**: CACCACCACCACCACCAC<br>**REV**: AAAAAACCTCCTTACTTTCTAGTCTCA AG	pET28b-RQF (Dr. Tobias Pirzer, Technical University of Munich)
	MBP	**FWD**: TCTTGAGACTAGAAAGTAAGGAGG TTTTTTATGCACCACCACCACCACCAC ggttcaggcATCGAAGAAGGTAAACTGGTAATC<br>**REV**: ATAGCCGGTCACgcctgaaccacctcccga AGTCTGCGCGTCTTTCAG	pET28b-His-MBP-SUMO (Dr. Christopher Lima, Sloan Kettering Institute)
	SmBiT-SpyTag	**IDT duplex oligo**: tcgggaggtggttcaggcGTGACCGGCTATCG CCTGTTTGAAGAAATTCTGgcgggttcaggcggttc aggccgtggcgttcctcatattgttatggtggacgcctacaaac gctataaaTGAGATCCGGCTGCTAACAAAGCC CGAAAG	N/A
pET28b-His-MBP-SmBiT	MBP-SmBiT	**Fragment made by 2-step PCR:**<br>**FWD1:** TCTTGAGACTAGAAAGTAAGGAGGTTTTTTA TGCACCACCACCACCACCACggttcaggcATC GAAGAAGGTAAACTGGTAATC<br>**REV1:** cgcCAGAATTTCTTCAAACAGGCGATAGCCG GTCACgcctgaaccacctcccgaAGTCTGCGCGTC TTTCAG<br>**FWD2:** TCTTGAGACTAGAAAGTAAGGAGGT<br>**REV2:** TTCCTTTCGGGCTTTGTTAGCAGCC GGATCTCACGCCAGAATTTCTTCAAACAGG	pET28b-His-MBP-SUMO (Dr. Christopher Lima, Sloan Kettering Institute)
pET28b-SpyCatch	SpyCatcher-LgBiT	**FWD:** TCTTGAGACTAGAAAGTAAGGAGG TTTTTTATGACCACACTGTCCGGACTG	pcDNA-xC-LgBiT (Dr. Taekjip Ha,

er-LgBiT-His		**REV:** AGCCGGATCTCAGTGGTGGTGGTGGT GGTGGCTGTTGATGGTTACTCGGAAC	Harvard Medical School)
pET28b-iLID-MBP-His	MBP	**FWD**: TCTTGAGACTAGAAAGTAAGGAGG TTTTTTatgATCGAAGAAGGTAAACTGGT **REV**: tgccagtccggatccagagccggagccgccT CCACCAATCTGTTCTCTG	pET28b-His-MBP-SUMO (Dr. Christopher Lima, Sloan Kettering Institute)
	iLID	**FWD**: GGTGgaggcggctccggctctggatccgga CTGGCAACCACACTGGAAC **REV**: AGCCGGATCTCAGTGGTGGTGGTG GTGGTGtcccgaaaagtaattttcgtcgttcgctgc	pHR-SFFVp-iLID::EGFP::FTH 1 (Dr. Kristin Verhey, University of Michigan)
pET28b-NLuc-MBP-His	MBP	**FWD**: TCTTGAGACTAGAAAGTAAGGAGG TTTTTTATGGTCTTCACACTCGAAGATTTCG **REV**: gcctgaaccacctcccgacgcAGAACCG CTCAGAATCTCCTC	pET28b-His-MBP-SUMO (Dr. Christopher Lima, Sloan Kettering Institute)
	NLuc	**FWD**: agcggttctgcgtcgggaggtggttcaggc ATCGAAGAAGGTAAACTGGTAATC **REV**: AGCCGGATCTCAGTGGTGGTGGTG GTGGTGGTGGTGGTGGTGtcccgaAGTCTGC GCGTCTTTCAG	pCMV-CXCL12-NLuc (Dr. Gary Luker, University of Michigan)
pGEX-mCherry-SspB	Vector	**FWD**: tagTGACTGACTGACGATCTGCCT **REV**: GGGCCCCTGGAACAGAA	pGEX6p1-Fascin-sfGFP (homemade)
	SspB-mCherry	**FWD**: TCGGATCTGGAAGTTCTGTTCCAGG GGCCCATGGTGTCTAAAGGCGAGG **REV**: CGCGCGAGGCAGATCGTCAGTCAGT CActaACCAATATTCAGCTCGTCATAG	pHR-SFFVp-FUSN::mCherry::SspB (Dr. Kristin Verhey, University of Michigan)

Tables S2: List of *p*-values:

Figure	Comparison groups	*p*-value
2-c	ST-SmBiT+SC-LgBiT vs. SmBiT+SC-LgBiT	0.00015
3-e	Light vs. Dark	1.11×10^{-12}
	Light vs. SspB	1.07×10^{-12}
	Dark vs. SspB	0.86
4-c	-Furimazine vs. +Furimazine	2.21×10^{-12}
4-f	-Furimazine vs. +Furimazine	2.18×10^{-12}
5-c	Iso-Furimazine vs. Iso+Furimazine	0.93
	Iso-Furimazine vs. Hyper+Furimazine	2.10×10^{-12}
	Hyper-Furimazine vs. Iso+Furimazine	0.83
	Hyper+Furimazine vs. Hyper-Furimazine	3.45×10^{-12}
	Hyper+Furimazine vs. Iso+Furimazine	1.25×10^{-12}
S1-c	1000 nM NLuc vs. 1000 nM NanoBiT	0.00032
	500 nM NLuc vs. 500 nM NanoBiT	0.0024
	100 nM NLuc vs. 100 nM NanoBiT	0.028
	50 nM NLuc vs. 50 nM NanoBiT	0.052
S5-b	+NLuc+NTA (Ni) vs. -NLuc+NTA (Ni)	1.05×10^{-12}
	+NLuc+NTA (Ni) vs. +NLuc-NTA (Ni)	1.05×10^{-12}
	+NLuc-NTA (Ni) vs. -NLuc+NTA (Ni)	0.97
S6	External light vs. NLuc inside	2.07×10^{-5}
	External light vs. NLuc outside	5.97×10^{-6}
	NLuc inside vs. NLuc outside	0.06
S8-d	-SpyTag-Furimazine vs. -SpyTag+Furimazine	0.08
	+SpyTag+Furimazine vs. -SpyTag-Furimazine	7.30×10^{-12}
	+SpyTag+Furimazine vs. -SpyTag+Furimazine	7.32×10^{-12}
	+SpyTag+Furimazine vs. +SpyTag-Furimazine	7.30×10^{-12}
	-SpyTag+Furimazine vs. +SpyTag-Furimazine	0.25
S9-b	+SpyTag vs. -SpyTag	6.50×10^{-5}

References:

1. Bashirzadeh, Y. *et al.* Actin crosslinker competition and sorting drive emergent GUV size-dependent actin network architecture. *Communications Biology 2021 4:1* **4**, 1–11 (2021).

2. Bashirzadeh, Y., Moghimianavval, H. & Liu, A. P. Encapsulated actomyosin patterns drive cell-like membrane shape changes. *iScience* **25**, 104236 (2022).

3. Moghimianavval, H. *et al.* Engineering Functional Membrane–Membrane Interfaces by InterSpy. *Small* **19**, 2202104 (2023).

4. Moghimianavval, H., Mohapatra, S. & Liu, A. P. A Mammalian-Based Synthetic Biology Toolbox to Engineer Membrane-Membrane Interfaces. *Methods Mol Biol* **2774**, 43–58 (2024).

5. Guntas, G. *et al.* Engineering an improved light-induced dimer (iLID) for controlling the localization and activity of signaling proteins. *Proc Natl Acad Sci U S A* **112**, 112–117 (2015).
